# Worldwide comparison of survival from childhood leukaemia for 1995–2009, by subtype, age, and sex (CONCORD-2): a population-based study of individual data for 89 828 children from 198 registries in 53 countries

**DOI:** 10.1016/S2352-3026(17)30052-2

**Published:** 2017-04-11

**Authors:** Audrey Bonaventure, Rhea Harewood, Charles A Stiller, Gemma Gatta, Jacqueline Clavel, Daniela C Stefan, Helena Carreira, Devon Spika, Rafael Marcos-Gragera, Rafael Peris-Bonet, Marion Piñeros, Milena Sant, Claudia E Kuehni, Michael F G Murphy, Michel P Coleman, Claudia Allemani, CONCORD Working Group, S Bouzbid, M Hamdi-Chérif, Z Zaidi, E Bah, R Swaminathan, SH Nortje, MM El Mistiri, S Bayo, B Malle, SS Manraj, R Sewpaul-Sungkur, Fabowale, OJ Ogunbiyi, D Bradshaw, NIM Somdyala, DC Stefan, M Abdel-Rahman, L Jaidane, M Mokni, I Kumcher, F Moreno, MS González, EA Laura, SB Espinola, GH Calabrano, B Carballo Quintero, R Fita, DA Garcilazo, PL Giacciani, MC Diumenjo, WD Laspada, MA Green, MF Lanza, SG Ibañez, CA Lima, E Lobo de Oliveira, C Daniel, C Scandiuzzi, PCF De Souza, CD Melo, K Del Pino, C Laporte, MP Curado, JC de Oliveira, CLA Veneziano, DB Veneziano, TS Alexandre, AS Verdugo, G Azevedo e Silva, JC Galaz, JA Moya, DA Herrmann, S Vargas, VM Herrera, CJ Uribe, LE Bravo, NE Arias-Ortiz, DM Jurado, MC Yépez, YH Galán, P Torres, F Martínez-Reyes, ML Pérez-Meza, L Jaramillo, R Quinto, P Cueva, JG Yépez, CR Torres-Cintrón, G Tortolero-Luna, R Alonso, E Barrios, C Nikiforuk, L Shack, AJ Coldman, RR Woods, G Noonan, D Turner, E Kumar, B Zhang, FR McCrate, S Ryan, H Hannah, RAD Dewar, M MacIntyre, A Lalany, M Ruta, L Marrett, DE Nishri, C McClure, KA Vriends, C Bertrand, R Louchini, KI Robb, H Stuart-Panko, S Demers, S Wright, JT George, X Shen, JT Brockhouse, DK O'Brien, KC Ward, L Almon, J Bates, R Rycroft, L Mueller, C Phillips, H Brown, B Cromartie, AG Schwartz, F Vigneau, JA MacKinnon, B Wohler, AR Bayakly, CA Clarke, SL Glaser, D West, MD Green, BY Hernandez, CJ Johnson, D Jozwik, ME Charlton, CF Lynch, B Huang, TC Tucker, D Deapen, L Liu, MC Hsieh, XC Wu, K Stern, ST Gershman, RC Knowlton, J Alverson, GE Copeland, DB Rogers, D Lemons, LL Williamson, M Hood, GM Hosain, JR Rees, KS Pawlish, A Stroup, C Key, C Wiggins, AR Kahn, MJ Schymura, G Leung, C Rao, L Giljahn, B Warther, A Pate, M Patil, SS Schubert, JJ Rubertone, SJ Slack, JP Fulton, DL Rousseau, TA Janes, SM Schwartz, SW Bolick, DM Hurley, J Richards, MA Whiteside, LM Nogueira, K Herget, C Sweeney, J Martin, S Wang, DG Harrelson, MB Keitheri Cheteri, S Farley, AG Hudson, R Borchers, L Stephenson, JR Espinoza, HK Weir, BK Edwards, N Wang, L Yang, JS Chen, GH Song, XP Gu, P Zhang, HM Ge, DL Zhao, JH Zhang, FD Zhu, JG Tang, Y Shen, J Wang, QL Li, XP Yang, J Dong, W Li, LP Cheng, JG Chen, QH Huang, SQ Huang, GP Guo, K Wei, WQ Chen, H Zeng, AV Demetriou, P Pavlou, WK Mang, KC Ngan, R Swaminathan, AC Kataki, M Krishnatreya, PA Jayalekshmi, P Sebastian, SD Sapkota, Y Verma, A Nandakumar, E Suzanna, L Keinan-Boker, BG Silverman, H Ito, H Nakagawa, M Hattori, Y Kaizaki, H Sugiyama, M Utada, K Katayama, H Narimatsu, S Kanemura, T Koike, I Miyashiro, M Yoshii, I Oki, A Shibata, T Matsuda, O Nimri, A Ab Manan, N Bhoo Pathy, O Chimedsuren, S Tuvshingerel, AHM Al Khater, MM El Mistiri, H Al-Eid, KW Jung, YJ Won, CJ Chiang, MS Lai, K Suwanrungruang, S Wiangnon, K Daoprasert, D Pongnikorn, SL Geater, H Sriplung, S Eser, CI Yakut, M Hackl, H Mühlböck, W Oberaigner, AA Zborovskaya, OV Aleinikova, K Henau, L Van Eycken, N Dimitrova, Z Valerianova, M Šekerija, M Zvolský, G Engholm, H Storm, K Innos, M Mägi, N Malila, K Seppä, J Jégu, M Velten, E Cornet, X Troussard, AM Bouvier, J Faivre, AV Guizard, V Bouvier, G Launoy, P Arveux, M Maynadié, M Mounier, E Fournier, AS Woronoff, M Daoulas, J Clavel, S Le Guyader-Peyrou, A Monnereau, B Trétarre, M Colonna, A Cowppli-Bony, F Molinié, S Bara, D Degré, O Ganry, B Lapôtre-Ledoux, P Grosclaude, J Estève, F Bray, M Piñeros, F Sassi, R Stabenow, A Eberle, C Erb, A Nennecke, J Kieschke, E Sirri, H Kajueter, K Emrich, SR Zeissig, B Holleczek, N Eisemann, A Katalinic, H Brenner, RA Asquez, V Kumar, EJ Ólafsdóttir, L Tryggvadóttir, H Comber, PM Walsh, H Sundseth, E Devigili, G Mazzoleni, A Giacomin, F Bella, M Castaing, A Sutera, G Gola, S Ferretti, D Serraino, A Zucchetto, R Lillini, M Vercelli, S Busco, F Pannozzo, S Vitarelli, P Ricci, C Pascucci, M Autelitano, C Cirilli, M Federico, M Fusco, MF Vitale, M Usala, R Cusimano, W Mazzucco, M Michiara, P Sgargi, MM Maule, C Sacerdote, R Tumino, E Di Felice, M Vicentini, F Falcini, L Cremone, M Budroni, R Cesaraccio, ML Contrino, F Tisano, AC Fanetti, S Maspero, G Candela, T Scuderi, MA Gentilini, S Piffer, S Rosso, L Sacchetto, A Caldarella, F La Rosa, F Stracci, P Contiero, G Tagliabue, AP Dei Tos, M Zorzi, R Zanetti, P Baili, F Berrino, G Gatta, M Sant, R Capocaccia, R De Angelis, E Liepina, A Maurina, G Smailyte, D Agius, N Calleja, S Siesling, O Visser, S Larønningen, B Møller, A Dyzmann-Sroka, M Trojanowski, S Gózdz, R Mezyk, M Gradalska-Lampart, AU Radziszewska, JA Didkowska, U Wojciechowska, J Blaszczyk, K Kepska, M Bielska-Lasota, K Kwiatkowska, G Forjaz, RA Rego, J Bastos, MA Silva, L Antunes, MJ Bento, A Mayer-da-Silva, A Miranda, D Coza, AI Todescu, MY Valkov, J Adamcik, C Safaei Diba, M Primic-Žakelj, T Žagar, J Stare, E Almar, A Mateos, JR Quirós, J Bidaurrazaga, N Larrañaga, JM Díaz García, AI Marcos, R Marcos-Gragera, ML Vilardell Gil, E Molina, MJ Sánchez, P Franch Sureda, M Ramos Montserrat, MD Chirlaque, C Navarro, EE Ardanaz, CC Moreno-Iribas, R Fernández-Delgado, R Peris-Bonet, J Galceran, S Khan, M Lambe, B Camey, C Bouchardy, M Usel, SM Ess, C Herrmann, JL Bulliard, M Maspoli-Conconi, H Frick, CE Kuehni, M Schindler, A Bordoni, A Spitale, A Chiolero, I Konzelmann, SI Dehler, KL Matthes, J Rashbass, CA Stiller, D Fitzpatrick, A Gavin, F Bannon, RJ Black, DH Brewster, DW Huws, C White, P Finan, C Allemani, A Bonaventure, H Carreira, MP Coleman, V Di Carlo, R Harewood, K Liu, M Matz, L Montel, M Nikšic, B Rachet, N Sanz, D Spika, R Stephens, M Peake, MFG Murphy, E Chalker, L Newman, D Baker, MJ Soeberg, J Aitken, C Scott, BC Stokes, A Venn, H Farrugia, GG Giles, T Threlfall, D Currow, H You, J Hendrix, C Lewis, MRDO Latorre, LF Tanaka

**Affiliations:** a Cancer Survival Group, Department of Non-Communicable Disease Epidemiology, London School of Hygiene & Tropical Medicine, London, UK; b National Cancer Registration and Analysis Service, Public Health England, Oxford, UK; c Evaluative Epidemiology Unit, Fondazione IRCCS Istituto Nazionale dei Tumori, Milan, Italy; d National Registry of Childhood Haematopoietic Malignancies, INSERM, Université Paris-Descartes, Université Sorbonne-Paris-Cité, CRESS-EPICEA Epidémiologie des Cancers de l'Enfant et de l'Adolescent, Paris, France; e Umtata University, Mthatha, South Africa; f Epidemiology Unit and Girona Cancer Registry, Oncology Coordination Plan, Department of Health, Catalan Institute of Oncology-Girona, Girona, Spain; g Registro Español de Tumores Infantiles, UVEG, Valencia, Spain; h Section of Cancer Surveillance, International Agency for Research on Cancer, Lyon, France; i Analytical Epidemiology and Health Impact Unit, Department of Preventive and Predictive Medicine, Fondazione IRCCS Istituto Nazionale dei Tumori, Milan, Italy; j Swiss Childhood Cancer Registry, Institute of Social and Preventive Medicine, University of Bern, Bern, Switzerland; k Nuffield Department of Obstetrics and Gynaecology, University of Oxford, Oxford, UK

## Abstract

**Background:**

Global inequalities in access to health care are reflected in differences in cancer survival. The CONCORD programme was designed to assess worldwide differences and trends in population-based cancer survival. In this population-based study, we aimed to estimate survival inequalities globally for several subtypes of childhood leukaemia.

**Methods:**

Cancer registries participating in CONCORD were asked to submit tumour registrations for all children aged 0–14 years who were diagnosed with leukaemia between Jan 1, 1995, and Dec 31, 2009, and followed up until Dec 31, 2009. Haematological malignancies were defined by morphology codes in the International Classification of Diseases for Oncology, third revision. We excluded data from registries from which the data were judged to be less reliable, or included only lymphomas, and data from countries in which data for fewer than ten children were available for analysis. We also excluded records because of a missing date of birth, diagnosis, or last known vital status. We estimated 5-year net survival (ie, the probability of surviving at least 5 years after diagnosis, after controlling for deaths from other causes [background mortality]) for children by calendar period of diagnosis (1995–99, 2000–04, and 2005–09), sex, and age at diagnosis (<1, 1–4, 5–9, and 10–14 years, inclusive) using appropriate life tables. We estimated age-standardised net survival for international comparison of survival trends for precursor-cell acute lymphoblastic leukaemia (ALL) and acute myeloid leukaemia (AML).

**Findings:**

We analysed data from 89 828 children from 198 registries in 53 countries. During 1995–99, 5-year age-standardised net survival for all lymphoid leukaemias combined ranged from 10·6% (95% CI 3·1–18·2) in the Chinese registries to 86·8% (81·6–92·0) in Austria. International differences in 5-year survival for childhood leukaemia were still large as recently as 2005–09, when age-standardised survival for lymphoid leukaemias ranged from 52·4% (95% CI 42·8–61·9) in Cali, Colombia, to 91·6% (89·5–93·6) in the German registries, and for AML ranged from 33·3% (18·9–47·7) in Bulgaria to 78·2% (72·0–84·3) in German registries. Survival from precursor-cell ALL was very close to that of all lymphoid leukaemias combined, with similar variation. In most countries, survival from AML improved more than survival from ALL between 2000–04 and 2005–09. Survival for each type of leukaemia varied markedly with age: survival was highest for children aged 1–4 and 5–9 years, and lowest for infants (younger than 1 year). There was no systematic difference in survival between boys and girls.

**Interpretation:**

Global inequalities in survival from childhood leukaemia have narrowed with time but remain very wide for both ALL and AML. These results provide useful information for health policy makers on the effectiveness of health-care systems and for cancer policy makers to reduce inequalities in childhood cancer survival.

**Funding:**

Canadian Partnership Against Cancer, Cancer Focus Northern Ireland, Cancer Institute New South Wales, Cancer Research UK, US Centers for Disease Control and Prevention, Swiss Re, Swiss Cancer Research foundation, Swiss Cancer League, and the University of Kentucky.

## Introduction

Worldwide inequalities in health and health care are reflected in global differences in life expectancy and overall mortality in both adults and children,^1^ and the findings from several studies have highlighted global differences in cancer incidence^2^ and survival.^3^, ^4^ Diagnostic techniques and treatment for childhood leukaemia have improved since the 1990s. Access to these techniques and treatment has, however, been limited in some countries, partly by a shortage of resources.^5^

Leukaemias, a heterogeneous group of diseases of mostly unknown origin, are globally the most common malignancies in children (aged 0–14 years), except in Africa.^6^ Unlike in adults, acute lymphoid leukaemias are the commonest subtype in children (accounting for approximately 80% of cases), and acute myeloid leukaemia (AML) represents about 15% of cases. For both types, incidence varies widely with age; in lymphoid leukaemias, incidence is slightly higher in boys than girls, and in industrialised high-income countries (HIC).^7^ In low-income and middle-income countries (LMIC), where the population is young, the incidence of childhood leukaemias is lower than in HIC, but these diseases are still responsible for many deaths.^2^, ^5^

**Research in context**

**Evidence before this study**

In 2015, the CONCORD-2 study initiated surveillance of survival trends for childhood leukaemia at a worldwide scale. Results from CONCORD-2 identified huge worldwide variation in 5-year net survival for children diagnosed with precursor-cell acute lymphoblastic leukaemia or lymphoma (ALL) during 1995-2009. Our analysis extends those results to cover survival trends for several subtypes of childhood leukaemia, grouped according to the third edition of the International Classification of Childhood Cancer.

**Added value of this study**

We included 89 828 children diagnosed with leukaemia during 1995–2009 in 53 countries. Despite substantial improvements in survival from childhood acute myeloid leukaemia (AML) in most countries during 1995–2009, huge international disparities in 5-year survival persisted up to 2009, matching those previously reported in the 2015 CONCORD-2 paper for childhood ALL. 5-year age-standardised net survival from AML (ie, the probability of surviving at least 5 years after diagnosis) was consistently lower than 5-year age-standardised net survival from ALL, but the difference narrowed in most countries since the early 2000s. Children aged 1–9 years at diagnosis had higher 5-year net survival than older or younger children, both for ALL and AML. Survival for older children (10–14 years) improved by 2009, but infants (aged <1 year) diagnosed with either ALL or AML still had the lowest 5-year net survival.

**Implications of all the available evidence**

Data obtained in the CONCORD programme provide a unique opportunity to explore disparities in survival from childhood leukaemia at an unprecedented scale. The results suggest that good access to health care and appropriate treatment have a clear population effect on survival for children with leukaemia. The findings support the need for continuing international efforts to improve worldwide access to appropriate cancer care for children. They can also be used to assess the effect of cancer strategies targeting childhood cancers.

Although cancer mortality trends provide a useful measure of the societal cancer burden, they depend on trends in both incidence and survival. Cancer survival is the probability that cancer patients survive up to a certain point after diagnosis. Observed survival and event-free survival are clinically important, but population-based net survival is the appropriate indicator for comparisons between populations. Net survival is the probability of surviving after controlling for mortality from other causes.

The CONCORD programme was designed to address the shortage of globally comparable data on population-based cancer survival.^3^, ^4^ Population-based cancer survival reflects several aspects of health care, from screening and early diagnosis, to access to effective treatment. This metric is increasingly used as a measure of the effectiveness of health-care systems in the management of cancer, and to assess the effectiveness of national cancer plans.^8^, ^9^ The second cycle of CONCORD (CONCORD-2)^4^ established global surveillance of population-based cancer survival for patients diagnosed with one of ten common cancers, or childhood leukaemia, during the 15-year period 1995–2009, using data from 279 population-based cancer registries in 67 countries. In this analysis, we examine worldwide trends in survival from precursor-cell acute lymphoblastic leukaemia (ALL) in children, by age and sex, alongside trends in survival from acute myeloid leukaemia (AML) and other types of childhood leukaemia.

## Methods

### Search strategy and selection criteria

Cancer registries participating in CONCORD-2 were asked to submit tumour registrations for all children (aged 0–14 years) diagnosed with a haematological malignancy between Jan 1, 1995, and Dec 31, 2009, including information about their vital status at Dec 31, 2009.^4^ Depending on the registry, patients were followed up actively, via direct investigation, or passively, using linkage to national or regional databases of death.^4^ Haematological malignancies were defined by morphology codes in the range 9590–9989 in the International Classification of Diseases for Oncology, third revision (ICD-O-3).^10^ 215 registries in 60 countries submitted data on 126 830 children with a haematological malignancy. We excluded data from 13 registries from which the data were judged to be less reliable,^4^ or included only lymphomas (two), and data from countries for which fewer than 10 children were available for analysis (two). This left 124 015 records for children with a haematological malignancy.

We did standardised data cleaning in three phases, as detailed previously.^4^ Records that were ineligible (eg, for patients aged 15 years or older), inaccurate or inappropriate for survival analysis (eg, incoherent date sequence, or registration only from a death certificate or autopsy report) were excluded.^4^, ^11^ For patients with more than one record of a haematological malignancy diagnosed during 1995–2009, we kept only the record of the first malignancy. A few registries submitted records coded to earlier revisions of ICD-O or to the first revision of ICD-O-3. In agreement with these registries, we recoded those morphology codes to be compliant with ICD-O-3. Data from Sétif (Algeria), Arkhangelsk (Russia), Wrocław (Poland), and Northern Ireland (UK) were included after their data were recoded to ICD-O-3.

### Data analysis

We estimated 5-year net survival with the Pohar-Perme estimator^12^ using the STNS command implemented in Stata 13. We used the life tables of background mortality rates by sex, single year of age, calendar year and—for USA, Israel, Malaysia, and New Zealand—by race or ethnic group, produced for CONCORD-2.^13^ For each country, we estimated net survival by calendar period of diagnosis (1995–99, 2000–04, and 2005–09). We used the classic cohort approach for children diagnosed during 1995–99 and 2000–04, because 5 years of follow-up data were available for all children. We used the period approach to predict 5-year survival for leukaemias diagnosed more recently (2005–09), as this approach allows for the prediction of survival where 5 years of follow-up are not yet available.^14^

Survival estimates for each country were based on data from a national registry or from one or several subnational registries. We excluded data from some regional registries from the pooled estimate for a given country if data quality or information about vital status were deemed unsatisfactory.^4^ Country estimates were flagged if data quality was considered less reliable.

We estimated 5-year survival by sex and age at diagnosis (<1, 1–4, 5–9, 10–14 years, inclusive). Exact age at diagnosis was calculated from the dates of birth and diagnosis. The rules adopted to impute missing components of dates have been described previously.^4^, ^11^ Age-standardised survival was calculated from three equally weighted age-specific estimates (0–4, 5–9, and 10–14 years).^15^ Data for age groups with fewer than ten patients were pooled with data for the adjacent age group; we then re-estimated survival for both age groups combined, and the pooled estimate was attributed to each age group.

Leukaemias were grouped according to the International Classification of Childhood Cancer (ICCC-3).^16^ We estimated survival for all lymphoid leukaemias combined (ICCC-3 group Ia), for acute myeloid leukaemias (AML; Ib), and for unspecified and other specified leukaemias (Ie) (appendix p 5). We also estimated survival separately for two subgroups of lymphoid leukaemia: precursor-cell lymphoid leukaemias (ALL; Ia1) and mature B-cell leukaemias (Ia2). We did not analyse survival for chronic myeloproliferative diseases (group Ic) or myelodysplastic syndrome and other myeloproliferative diseases (group Id).

Ethical approval for access to the data was obtained from the Ethics and Confidentiality Committee of the UK's statutory National Information Governance Board (now the Health Research Authority; ECC 3-04(i)/2011) and the UK National Health Service (NHS) Research Ethics Service (South-East; 11/LO/0331), and from other jurisdictions as required.^4^

### Role of the funding source

The funders had no role in study design, data collection, data analysis, data interpretation, or writing of the report. The corresponding author had full access to all data in the study and had final responsibility for the decision to submit for publication.

## Results

Of the 124 015 children who were considered for analysis, we excluded 1623 (1%) as ineligible, usually because of missing information on date of birth, diagnosis, or last known vital status (table 1). More than 75% of records from the Tunisia Central Registry were ineligible because of incomplete data. Overall, only 0·5% of records were excluded because the registration was based solely on a death certificate or an autopsy report. We excluded five patients with synchronous leukaemia and lymphoma, and 106 children whose leukaemia followed a lymphoma, also diagnosed during 1995–2009. We also excluded 2222 children with chronic myeloproliferative disease (ICCC-3 group Ic) and 2002 children with myelodysplastic syndrome or other myeloproliferative disease (Id). Lymphomas (27 609) were not included.

Data quality was generally very high, and a high proportion of diagnoses were reported with a specific morphology. Improvements in diagnosis and registration are illustrated in Latvia by the drop in the number of unspecified and other specified leukaemias (group Ie) between 1995–99 and 2005–09.

We focused our analyses on 89 828 children (73·8% of all haematological malignancies) from 198 registries in 53 countries who were diagnosed with lymphoid leukaemia (ICCC-3 group Ia), acute myeloid leukaemia (Ib), or unspecified or other specified leukaemia (Ie).

For children diagnosed during 1995–2004 who were not known to have died, the median follow-up was at least 5 years in all participating countries in North, Central, and South America, Europe, Asia, and Oceania. In Africa, the median follow-up of surviving children diagnosed up to 2004 was 0·7 years (IQR 0·2–3·0) in the two Algerian registries and 1·7 years (1·0–2·2) in the Tunisia Central Registry, although the maximum follow-up was 8·9 and 13·3 years, respectively.

**Figure 1:**
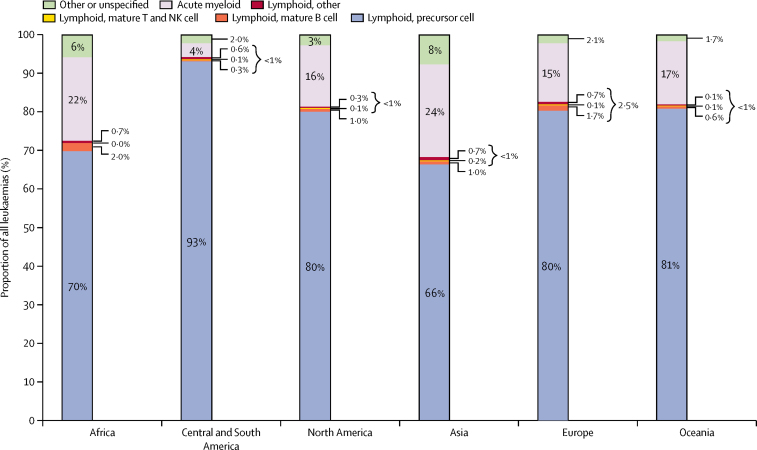
Distribution (%) of leukaemia subtypes in children diagnosed during 1995–2009 and included in survival analyses, by continent Leukaemias were classified according to the third edition of the International Classification of Childhood Cancer.

All lymphoid leukaemias combined represented 81% of leukaemias, AML 16%, and unspecified and other specified leukaemias the remaining 3% (table 1). In Lesotho, no AML was registered during 1995–2009. Information from the cancer registries in Belarus, Argentina, and Colombia (Cali) was only available for lymphoid leukaemias. Precursor-cell ALL was by far the most common type of lymphoid leukaemia, but some registries submitted data on rarer types, such as mature B-cell (mostly Burkitt leukaemia), mature T and Natural-Killer (NK) cell leukaemias (figure 1). Due to the rarity of mature T-cell and NK-cell leukaemia (n=94, ICCC-3 group Ia3) and unspecified lymphoid leukaemias (n=446, Ia4), we did not estimate survival separately for these morphological groups. In the data from Indonesia (Jakarta), 17 Chinese registries, and Latvia, more than 25% of the childhood leukaemias were coded as unspecified or other specified leukaemia (ICCC-3 group Ie).

**Figure 2:**
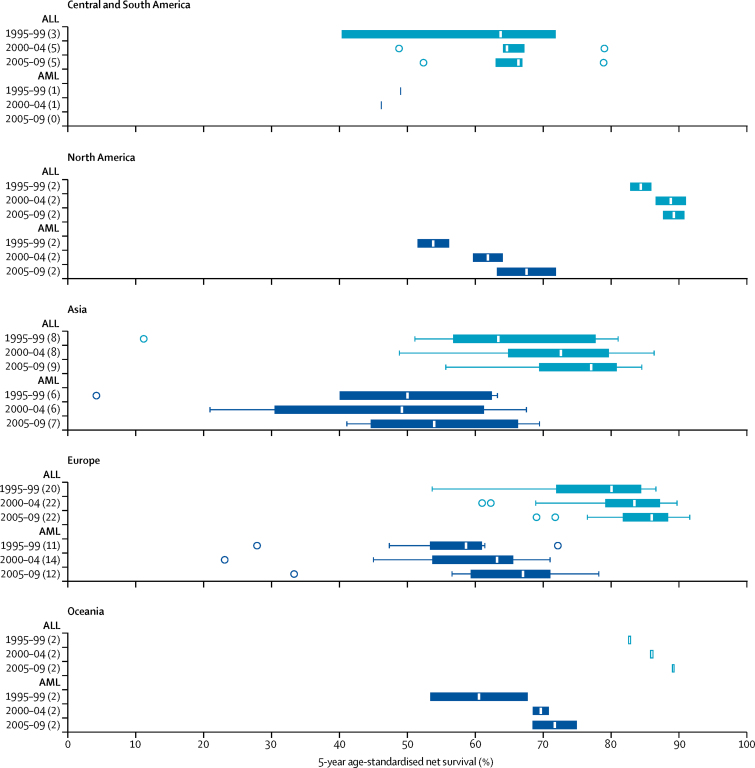
Age-standardised 5-year net survival (%) for children diagnosed with acute lymphoblastic leukaemia (ALL) and acute myeloid leukaemia (AML) during 1995–2009 The number of countries for which survival estimates are shown in each box-plot is given in parentheses. Box-plots in light blue are for ALL (group Ia1 according to the third edition of the International Classification of Childhood Cancer [ICCC-3]), and dark blue for AML (ICCC-3 group Ib). The vertical line inside each box denotes the median survival value, and the box shows the IQR between the lower and upper quartiles. The extreme limits of the box-plot are 1·5 times the IQR below the lower quartile and above the upper quartile. Open circles indicate outlier values, outside this range Survival estimates for African countries are not shown because they were either not standardised or less reliable.

**Figure 3:**
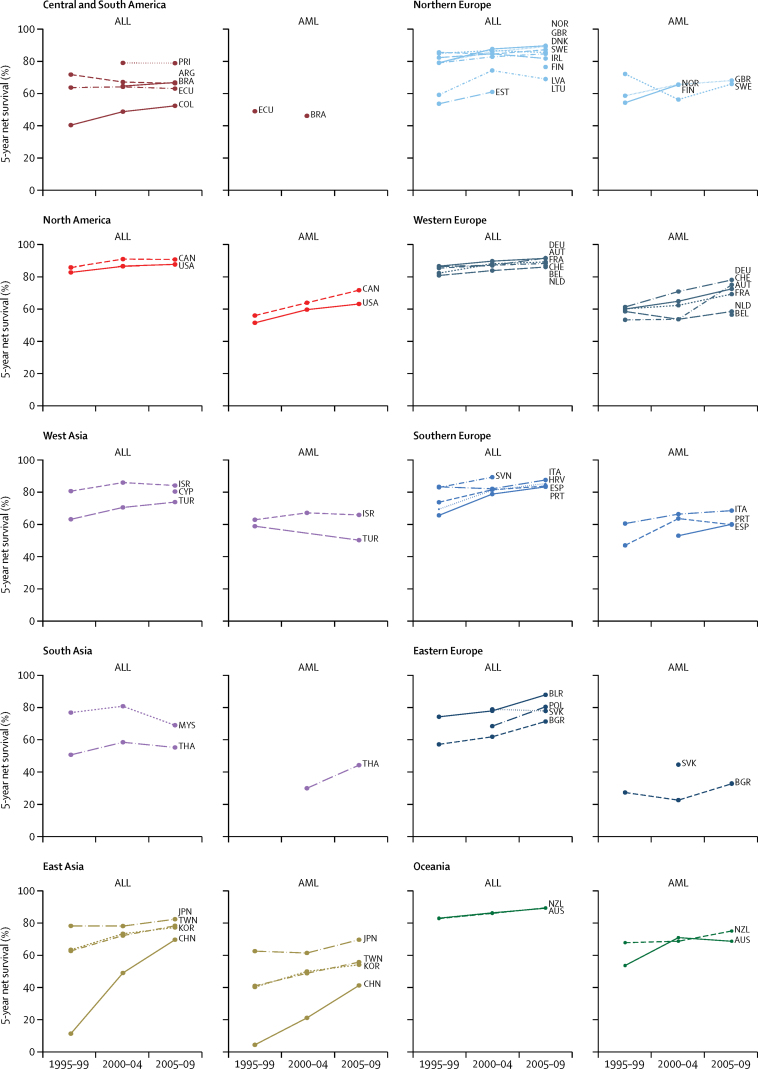
Trends in age-standardised 5-year net survival (%) for children diagnosed with acute lymphoblastic leukaemia (ALL) and acute myeloid leukaemia (AML), during 1995–1999, 2000–2004, and 2005–2009 Countries have been grouped into ten geographical regions. Survival estimates for African countries are not shown because they were either not standardised or less reliable. ALL: group Ia1 according to the third edition of the International Classification of Childhood Cancer (ICCC-3). AML: ICCC-3 group Ib. ARG=Argentina. AUS=Australia. AUT=Austria. BEL=Belgium. BGR=Bulgaria. BLR=Belarus. BRA=Brazil. CAN=Canada. CHE=Switzerland. CHN=China. COL=Colombia. CYP=Cyprus. DEU=Germany. DNK=Denmark. ECU=Ecuador. ESP=Spain. EST=Estonia. FIN=Finland. FRA=France. GBR=United Kingdom. HRV=Croatia. IRL=Ireland. ISR=Israel. ITA=Italy. JPN=Japan. KOR=Republic of Korea. LTU=Lithuania. LVA=Latvia. MYS=Malaysia. NLD=Netherlands. NOR=Norway. NZL=New Zealand. POL=Poland. PRI=Puerto Rico. PRT=Portugal. SVK=Slovakia. SVN=Slovenia. SWE=Sweden. TWN=Taiwan. THA=Thailand. TUR=Turkey. USA=United States of America.

During 1995–99, 5-year age-standardised net survival for all lymphoid leukaemias combined ranged from 10·6% (95% CI 3·1–18·2) in the Chinese registries to 86·8% (81·6–92·0) in Austria (table 2). This wide range in survival narrowed over time, with survival in 2005–09 ranging from 52·4% (42·8–61·9) in Cali, Colombia, to 91·6% (89·5–93·6) in the German registries. Survival from precursor-cell ALL was very close to that of all lymphoid leukaemias combined, with similar variation (figure 2). The greatest absolute difference in survival between all lymphoid leukaemias and ALL was noted in Iceland (80·9% and 90·1%, respectively) but these estimates were not age-standardised because of small numbers. Survival from precursor-cell ALL increased between 1995–99 and 2005–09 in most countries (figure 3).

We estimated survival for mature B-cell leukaemia for 17 countries. Many of these estimates were based on a pooled analysis for children diagnosed throughout 1995–2009, because the number of patients diagnosed in each 5-year period was small. In France and the US registries, 5-year age-standardised net survival from mature B-cell leukaemia was lower than that of precursor-cell ALL in 1995–99, but the difference had disappeared by 2005–09.

5-year age-standardised net survival for AML was consistently lower than that for ALL (table 2, figure 2). Age-standardised survival for AML in 1995–99 ranged from 4·2% (0·0–8·6) in the Chinese registries to 72·2% (61·6–82·7) in Sweden, and from 33·3% (18·9–47·7) in Bulgaria to 78·2% (72·0–84·3) in German registries for 2005–09. In most countries, survival from childhood AML increased quite remarkably over time (figure 3). Age-standardised 5-year survival for unspecified and other specified leukaemia (group Ie) showed wide variation, ranging from 13·5% (Bulgaria) to 69·6% (Latvia) in 1995–99, and from 26·0% (Chinese registries) to 89·2% (Israel) in 2005–09.

Survival from ALL in infants (aged <1 year) was much lower than for older children, including those aged 10–14 years (appendix pp 6–14). By contrast, survival for infants with AML in many countries was close to that for children aged 10–14 years (appendix pp 15–22). Children aged 1–4 and 5–9 years had the highest survival for both ALL and AML. The difference in survival between children with ALL aged 10–14 and those aged 1–4 and 5–9 years fell progressively between 1995–99 and 2005–09 in most countries. The pattern was not so clear for AML. Survival from AML and ALL was often slightly higher for girls than for boys, but this pattern was not consistent across all countries (appendix pp 6–22).

**Figure 4:**
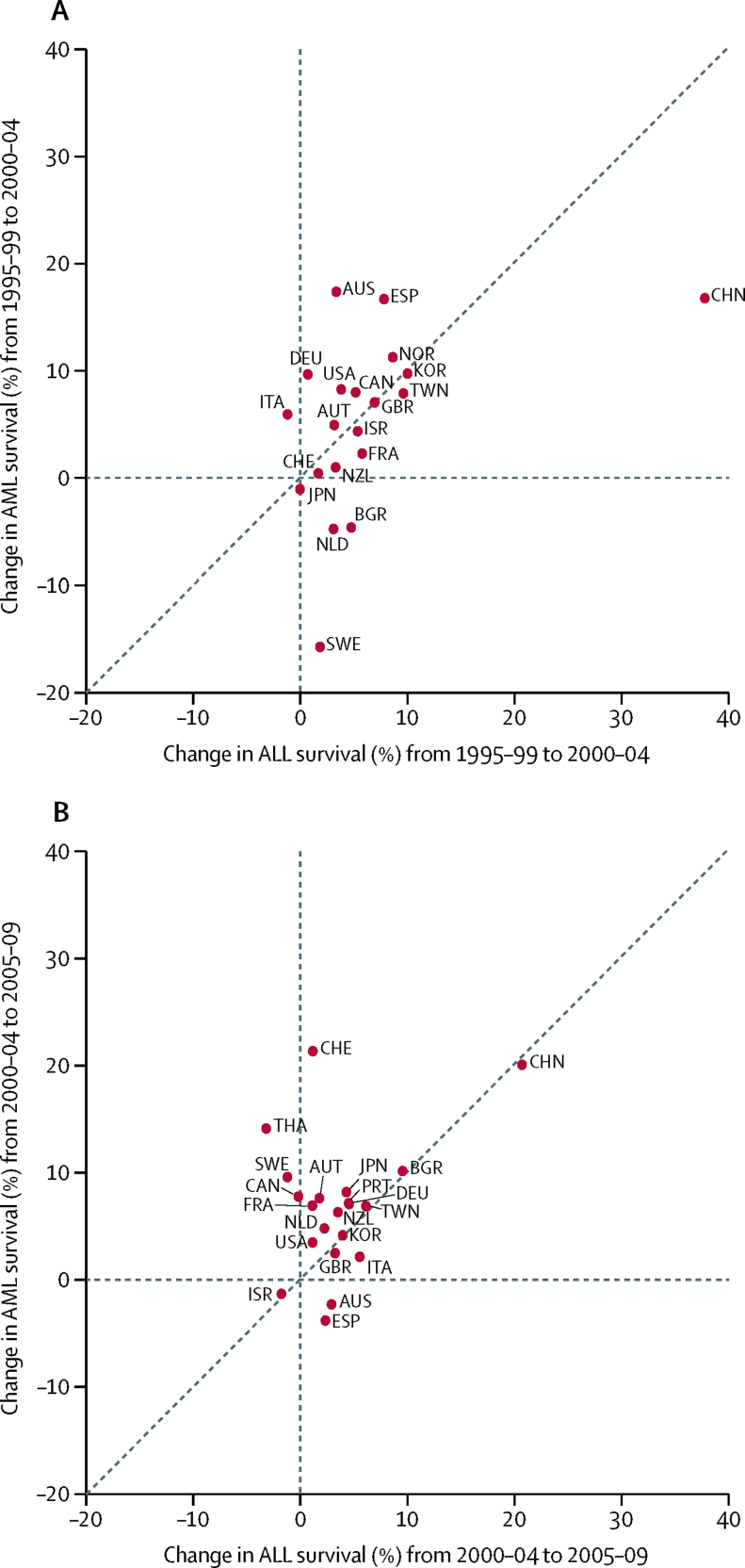
Change (absolute difference, %) in age-standardised 5-year net survival for acute lymphoblastic leukaemia (ALL) and acute myeloid leukaemia (AML), between (A) 1995–99 and 2000–04 and (B) between 2000–04 and 2005–09 Each datapoint represents one of the participating countries. Datapoints above the diagonal indicate that survival from AML increased more than survival from ALL between the two calendar periods. Countries are represented only if 5-year age-standardised estimates were available for ALL and for AML in successive calendar periods. ALL: group Ia1 according to the third edition of the International Classification of Childhood Cancer (ICCC-3). AML: ICCC-3 group Ib. AUS=Australia. AUT=Austria. BGR=Bulgaria. CAN=Canada. CHE=Switzerland. CHN=China. DEU=Germany. ESP=Spain. FRA=France. GBR=United Kingdom. ISR=Israel. ITA=Italy. JPN=Japan. KOR=Republic of Korea. NLD=Netherlands. NOR=Norway. NZL=New Zealand. PRT=Portugal. SWE=Sweden. THA=Thailand. TWN=Taiwan. USA=United States of America.

Survival trends for precursor-cell ALL and AML were not markedly different between 1995–99 and 2000–04 (figure 4); however, between 2000–04 and 2005–09, survival from AML increased more than survival from ALL in most countries (figure 4B), particularly in Thailand and Switzerland.

## Discussion

This study provides the largest population-based comparison of survival from childhood leukaemia. It covers trends over 15 years between 1995–2009 in 53 countries, of which 17 were classified by the World Bank as low-income or middle-income countries (LMIC) in 2011. The results highlight the very wide international differences in 5-year net survival for children with acute lymphoid leukaemia and for children with myeloid leukaemia. 5-year net survival has been increasing for both precursor-cell ALL and AML, but it remains consistently higher for precursor-cell ALL than for AML; this worldwide pattern is consistent with previous studies in various regions of the world.^7^, ^17^, ^18^, ^19^, ^20^ For many LMICs, analyses and interpretation are limited by sparse data. Several survival estimates could not be age-standardised. However, our results show that, overall, survival has been increasing for ALL and for AML in most countries during 1995–2009, including both high-income and LMICs.

5-year survival for both ALL and AML was very high in Germany and Austria. This might be attributable to the very tight adherence of paediatric haematologists and oncologists in those countries to the protocols and trials of the BFM Group (Berlin, Frankfurt, Muenster), within the framework of a national paediatric cancer registry and reference laboratories, imaging review and tumour boards.^21^

In most countries, the difference in survival between ALL and AML tended to narrow over the period 1995–2009. This happened both in countries where survival from ALL was very high throughout 1995–2009, and in countries where survival from ALL was low in 1995–99, but increased up to 2005–09. The greater improvement in 5-year survival from AML than from ALL might be related to recent improvements in the care of childhood AML. Our results might reflect the effect of better diagnostic characterisation, risk stratification and subsequent adaptation of treatment, and restriction of indications to cranial radiotherapy and haemopoietic stem-cell transplantation.^22^

Net survival from AML seemed to approach the level of survival from precursor-cell ALL earlier in some countries than in others. This suggests that improvements in clinical practice have not been implemented in all countries at the same time. In Switzerland, there was a large increase in survival from AML between 1995–2004 and 2005–09, by which time it approached the level of survival from ALL. In Thailand, national protocols were introduced for childhood leukaemia in 2006. This may have contributed to the marked improvement in survival from AML, but did not lead to substantial improvements in survival for ALL.^23^ In China, despite the impressive increases in survival from AML and ALL throughout 1995–2009, there was no reduction in the difference in survival between ALL and AML.

This large study offered a unique opportunity to examine age-standardised trends in population-based survival from some of the rarer childhood leukaemias, such as mature B-cell leukaemias, mostly Burkitt's leukaemia. In France and the USA, where survival from mature B-cell leukaemia could be age-standardised, the most recent estimates showed that survival from mature B-cell leukaemia was close to that of precursor-cell ALL. A degree of misclassification between precursor-cell ALL and Burkitt's leukaemia is likely because of their common historical classification and the remaining ambiguity in coding, but this is unlikely to explain the increasing survival trends for mature B-cell leukaemia in France and the USA.

Leukaemia survival has been reported as higher in girls than in boys, both in Europe from 1970 up to the 1990s,^24^ and still recently in North America,^18^ but this is not a consistent feature worldwide.

As expected, age at diagnosis was an important determinant of 5-year survival: children diagnosed with precursor-cell ALL aged 1–4 years consistently had the best prognosis. Survival from leukaemia in infants (under 1 year) was usually lower than at older ages. Survival was lower for infants with precursor-cell ALL than for infants with AML. This might be because unfavourable prognostic factors such as *MLL* gene rearrangements are more frequent in infants with ALL than AML. The new coding rules to individualise ALLs and AMLs with specific genetic rearrangements should enable analysis of more specific subgroups in the future.^25^

Interpretation might be restricted by changes in the clinical definition of leukaemias and lymphomas over time, and differences in coding between registries. The classifications for grouping the types of leukaemia and lymphoma have also changed over decades. ICCC-3 was proposed in 2005,^16^ and, in 2010, the HAEMACARE Working Group proposed a classification for haematological malignancies in both adults and children.^26^ Results shown here were based on ICCC-3, which is commonly used for childhood cancer surveillance.^7^, ^17^ Using the HAEMACARE classification, we noted similar results for precursor-cell ALL and AML, but some differences for Burkitt's leukaemia, probably due to the broader grouping defined by HAEMACARE (data not shown). We did not have data to show the effect on survival estimates of including endemic Burkitt's lymphoma in Africa in that group.

Children are more likely to be diagnosed and registered with a poorly specified type of leukaemia in settings where access to a pathologist is less than optimal. This could have clinical implications, with children not gaining access to the most appropriate treatment for their particular leukaemia.^27^ Where the estimates for this poorly specified group of leukaemias could be age-standardised, they indicate that in high-income countries, survival was between that of precursor-cell ALL and AML.

For the Chinese registries, where the proportion of unspecified and other specified leukaemias remained higher than 25% throughout 1995–2009, survival for those leukaemias was lower than that for AML. This suggests uncertain diagnosis and insufficient or inappropriate treatment. Implementation of the recent resource-stratified guidelines for the management of childhood ALL in Asia should lead to better diagnostic characterisation, more appropriate treatment and higher survival.^28^ In Colombia, the low survival estimate for lymphoid leukaemia (52%) suggests the need for further investigation, since childhood cancer became a national priority in 2010.

Leukaemia is the most common malignancy in children in most countries,^6^ and we used population-based data, but comparisons of survival by age and type of leukaemia were sometimes limited by low numbers, especially in small populations. Some age-standardised survival estimates have wide CIs. This was particularly true for AML and mature B-cell leukaemia, and for all types of leukaemia in infants. Survival estimates for Tunisian and Algerian registries were less reliable than for other countries. We used the age range 0–14 years for our analysis because this range has been the standard in childhood cancer studies for many years. Adolescents and young adults are increasingly being treated under paediatric protocols, but our choice was agreed with 100 collaborators during a 2-day meeting of the CONCORD Working Group (Cork, Ireland, 2012), at which the protocol was finalised. Detailed analyses of survival for adolescents, young adults, and older adults with leukaemia are under preparation for publication elsewhere.

Other studies of childhood cancer survival have not used net survival. The US SEER programme presents relative survival.^18^ The European programmes, EUROCARE^17^ and ACCIS,^7^ present observed survival, because background mortality in children does not vary greatly between European populations. The CONCORD-2 study has worldwide coverage, so we estimated net survival, because infant mortality varied particularly widely between participating countries.^4^ For example, infant mortality rates in males covered a 25-fold range in 2007, from 3·0 per 1000 livebirths in Finland to 82·5 per 1000 livebirths in Lesotho.^13^

Population-based cancer registry data include all or nearly all cases of a given malignancy in each registry's jurisdiction. By contrast with the best achievable survival estimates obtained from patients included in clinical trials, population-based survival estimates reflect the survival of all cancer patients in the population, irrespective of socioeconomic status and disease features. They reflect the overall effectiveness of the health system, from parents' perception of how to respond to symptoms suggestive of malignancy in their child, as well as the efficiency of referral, the quality of investigation and treatment, and the resourcing and organisation of the health service.

In many high-income countries, the incidence of ALL is rising by an average of about 1% every year.^7^, ^18^ Whether this is due to a true increase, improved registration, or both, is still debated.^29^ It is unlikely that improved diagnosis of the less aggressive forms of leukaemia can explain the very widespread rises in survival that we report. In many countries, childhood cancer treatment is provided in specialised centres, which makes cancer registration for children easier than for adults, although active follow-up can be more challenging in children and adolescents. Population-based cancer registry data might still be restricted by underdiagnosis and under-registration of cancer patients, both of which are difficult to quantify. This might be a more important issue for participating registries in the 17 LMICs, but it is nevertheless important to capture the available information as a guide to policy.

There is still room for improvement in the management of childhood leukaemia in many countries. In some LMICs, even the most basic treatment for leukaemia,^28^, ^30^ or pain relief, was still not consistently available until recently.^31^ Abandonment of treatment is also a major issue in some settings.^32^, ^33^ 5-year survival for children with precursor-cell lymphoblastic leukaemia can be as high as 90%, and up to 80% for children with AML, but in some countries, survival remains below 60% for both types of leukaemia. Interventions that have been proven to improve outcomes in childhood malignancy include enrolment in clinical trials, international collaboration, and treatment guidelines.^5^, ^34^, ^35^ Wider implementation of these initiatives, together with mobilisation of additional resources, especially in poorer countries, would be likely to improve the delivery of effective treatments, and to reduce worldwide inequalities in survival.^8^, ^9^, ^28^, ^36^, ^37^

## Figures and Tables

**Table 1:** Data quality indicators for children aged 0–14 years diagnosed with leukaemia between 1995–2009: by continent and country

		**Calendar period**	**Registries (n)**	**Patients submitted (n)**	**Eligible patients (n [%])**	**DCO (n [%])**	**Any haematological malignancy (n [% of eligible])**	**Leukaemia**					
								Included in analyses (n [% of any haematological malignancy])	Microscopically verified (n [%])	Follow-up time (years) of alive patients* (median [IQR])	Lymphoid leukaemia (n [%])	Acute myeloid leukaemia (n [%])	Unspecified and other specified leukaemia (n [%])
Africa		··	··	373	223 (60%)	1 (<1%)	193 (87%)	153 (79%)	152 (99%)	3·5 (0·5–4·7)	111 (73%)	33 (22%)	9 (6%)
	Algerian registries	2000–09	2	131	128 (98%)	0	99 (77%)	62 (63%)	61 (98%)	0·7 (0·2–3·0)	37 (60%)	20 (32%)	5 (8%)
	Lesotho†	1995–2009	1	27	27 (100%)	0	27 (100%)	27 (100%)	27 (100%)	6·6 (0·9–8·4)	25 (93%)	0	2 (7%)
	Libya (Benghazi)	2003–04	1	23	23 (100%)	1 (4%)	22 (96%)	21 (95%)	21 (100%)	4·9 (0·0–5·2)	14 (67%)	5 (24%)	2 (10%)
	Tunisia (Central)	1996–2007	1	192	45 (23%)	0	45 (100%)	43 (96%)	43 (100%)	1·7 (1·0–2·2)	35 (81%)	8 (19%)	0
America (Central and South)		··	··	5731	5722 (>99%)	175 (3%)	5488 (96%)	5086 (93%)	5063 (>99%)	9·2 (7·3–11·5)	4788 (94%)	195 (4%)	103 (2%)
	Argentina†	2000–09	1	3671	3671 (100%)	128 (3%)	3496 (95%)	3496 (100%)	3496 (100%)	6·5 (4·9–8·3)	3496 (100%)	0	0
	Brazilian registries	1996–2009	5	687	678 (99%)	41 (6%)	626 (92%)	497 (79%)	475 (96%)	11·2 (9·8–13·1)	387 (78%)	79 (16%)	31 (6%)
	Chilean registries	1998–2008	2	116	116 (100%)	0	116 (100%)	96 (83%)	96 (100%)	9·2 (7·7–12·4)	66 (69%)	14 (15%)	16 (17%)
	Colombia (Cali)	1995–2009	1	383	383 (100%)	0	382 (>99%)	381 (>99%)	381 (100%)	6·8 (3·6–9·2)	381 (100%)	0	0
	Ecuador (Quito)	1995–2009	1	507	507 (100%)	4 (1%)	503 (99%)	375 (75%)	375 (100%)	13·8 (11·5–17·3)	295 (79%)	67 (18%)	13 (3%)
	Puerto Rico†	2000–09	1	367	367 (100%)	2 (1%)	365 (99%)	241 (66%)	240 (>99%)	7·4 (6·2–8·6)	163 (68%)	35 (15%)	43 (18%)
America (North)		··	··	51 195	51 110 (>99%)	144 (<1%)	50 920 (>99%)	36 970 (73%)	36 156 (98%)	10·7 (8·3–13·3)	30 088 (81%)	5894 (16%)	988 (3%)
	Canada†	1995–2009	13	5259	5200 (99%)	13 (<1%)	5186 (>99%)	4152 (80%)	3983 (96%)	11·0 (8·6–13·5)	3363 (81%)	623 (15%)	166 (4%)
	US registries	1995–2009	38	45 936	45 910 (>99%)	131 (<1%)	45 734 (>99%)	32 818 (72%)	32 173 (98%)	10·3 (8·0–13·1)	26 725 (81%)	5271 (16%)	822 (3%)
Asia		··	··	17 371	17 176 (99%)	74 (<1%)	17 094 (>99%)	12 552 (73%)	12 309 (98%)	9·6 (7·4–11·8)	8553 (68%)	3051 (24%)	948 (8%)
	Chinese registries	1995–2009	17	741	728 (98%)	0	728 (100%)	632 (87%)	619 (98%)	7·1 (6·3–8·2)	309 (49%)	149 (24%)	174 (28%)
	Cyprus†	2004–09	1	49	49 (100%)	2 (4%)	47 (96%)	46 (98%)	46 (100%)	5·4 (5·1–5·8)	35 (76%)	10 (22%)	1 (2%)
	India (Karunagappally)	1995–2009	1	54	54 (100%)	0	54 (100%)	53 (98%)	53 (100%)	11·2 (7·4–14·8)	43 (81%)	8 (15%)	2 (4%)
	Indonesia (Jakarta)	2005–07	1	29	29 (100%)	0	28 (97%)	28 (100%)	26 (93%)	··	14 (50%)	6 (21%)	8 (29%)
	Israel†	1995–2009	1	1880	1775 (94%)	13 (1%)	1759 (99%)	1002 (57%)	997 (>99%)	10·3 (8·1–12·8)	731 (73%)	185 (18%)	86 (9%)
	Japanese registries	1995–2009	9	1729	1727 (>99%)	46 (3%)	1681 (97%)	1441 (86%)	1407 (98%)	10·1 (6·2–11·3)	969 (67%)	414 (29%)	58 (4%)
	Korea†‡	1995–2009	1	7569	7507 (99%)	0	7507 (100%)	5422 (72%)	5279 (97%)	11·5 (9·2–14·1)	3583 (66%)	1419 (26%)	420 (8%)
	Malaysia (Penang)	1995–2009	1	274	269 (98%)	3 (1%)	263 (98%)	261 (99%)	260 (>99%)	9·4 (7·6–11·9)	163 (62%)	67 (26%)	31 (12%)
	Mongolia†	2005–09	1	47	42 (89%)	0	41 (98%)	41 (100%)	31 (76%)	··	25 (61%)	8 (20%)	8 (20%)
	Taiwan†	1995–2009	1	3642	3642 (100%)	0	3642 (100%)	2641 (73%)	2612 (99%)	11·5 (9·2–14·0)	1935 (73%)	614 (23%)	92 (3%)
	Thai registries	1995–2009	3	537	537 (100%)	6 (1%)	531 (99%)	469 (88%)	463 (99%)	10·8 (7·8–12·8)	327 (70%)	85 (18%)	57 (12%)
	Turkey (Izmir)	1995–2009	1	820	817 (>99%)	4 (<1%)	813 (>99%)	516 (63%)	516 (100%)	9·2 (7·3–12·3)	419 (81%)	86 (17%)	11 (2%)
Europe		··	··	45 127	44 003 (98%)	165 (<1%)	43 815 (>99%)	31 797 (73%)	31 256 (98%)	11·0 (8·9–13·2)	26 262 (83%)	4856 (15%)	679 (2%)
	Austria†	1995–2009	2	1248	1209 (97%)	0	1203 (>99%)	826 (69%)	823 (>99%)	12·2 (9·5–14·4)	681 (82%)	122 (15%)	23 (3%)
	Belarus†	1995–2009	1	745	745 (100%)	0	745 (100%)	745 (100%)	744 (>99%)	12·6 (10·0–15·0)	745 (100%)	0	0
	Belgium†	2004–09	1	747	747 (100%)	0	747 (100%)	466 (62%)	465 (>99%)	8·0 (7·8–8·3)	396 (85%)	68 (15%)	2 (<1%)
	Bulgaria†	1995–2009	1	1079	1079 (100%)	0	1079 (100%)	714 (66%)	678 (95%)	13·0 (9·9–15·5)	558 (78%)	96 (13%)	60 (8%)
	Croatia†	1998–2009	1	651	651 (100%)	0	651 (100%)	446 (69%)	446 (100%)	9·9 (8·8–11·8)	375 (84%)	61 (14%)	10 (2%)
	Denmark†	1995–2009	1	695	695 (100%)	0	695 (100%)	680 (98%)	660 (97%)	10·4 (8·3–12·9)	551 (81%)	112 (16%)	17 (3%)
	Estonia†	1995–2008	1	160	160 (100%)	1 (1%)	159 (99%)	99 (62%)	99 (100%)	11·9 (9·1–14·4)	73 (74%)	24 (24%)	2 (2%)
	Finland†	1995–2009	1	1007	1007 (100%)	6 (1%)	1001 (99%)	728 (73%)	726 (>99%)	11·0 (8·5–13·5)	593 (81%)	101 (14%)	34 (5%)
	France†	1995–2009	1	10 619	10 032 (95%)	0	10 030 (>99%)	6875 (69%)	6873 (>99%)	9·4 (7·2–12·5)	5611 (82%)	1109 (16%)	155 (2%)
	German registries	1995–2009	13	3801	3781 (99%)	96 (3%)	3684 (97%)	2661 (72%)	2460 (92%)	10·7 (8·4–13·4)	2162 (81%)	436 (16%)	63 (2%)
	Iceland†	1995–2009	1	36	36 (100%)	0	36 (100%)	36 (100%)	36 (100%)	10·9 (10·1–13·0)	29 (81%)	6 (17%)	1 (3%)
	Ireland†	1995–2009	1	817	797 (98%)	2 (<1%)	794 (>99%)	571 (72%)	570 (>99%)	10·6 (8·2–13·2)	458 (80%)	99 (17%)	14 (2%)
	Italian registries	1995–2009	32	3955	3602 (91%)	10 (<1%)	3584 (>99%)	2313 (65%)	2247 (97%)	9·7 (7·3–12·6)	1930 (83%)	336 (15%)	47 (2%)
	Latvia†	1995–2009	1	205	205 (100%)	0	204 (>99%)	196 (96%)	194 (99%)	13·4 (11·5–16·6)	97 (49%)	35 (18%)	64 (33%)
	Lithuania†	1995–2009	1	532	529 (99%)	2 (<1%)	527 (>99%)	358 (68%)	358 (100%)	10·9 (9·1–13·6)	301 (84%)	53 (15%)	4 (1%)
	Malta†	1995–2009	1	92	92 (100%)	0	92 (100%)	61 (66%)	60 (98%)	10·8 (7·9–13·2)	53 (87%)	8 (13%)	0
	Netherlands†	1995–2009	1	2860	2858 (>99%)	3 (<1%)	2855 (>99%)	1988 (70%)	1988 (100%)	12·4 (10·1–15·0)	1652 (83%)	307 (15%)	29 (1%)
	Norway†	1995–2009	1	665	665 (100%)	1 (<1%)	664 (>99%)	646 (97%)	646 (100·0)	10·8 (7·9–13·3)	531 (82%)	108 (17%)	7 (1%)
	Poland (Wroclaw)	1995–2009	1	205	205 (100%)	0	203 (99%)	120 (59%)	120 (100%)	10·2 (9·2–11·4)	96 (80%)	24 (20%)	0
	Portugal†	1998–2009	4	872	855 (98%)	0	854 (>99%)	665 (78%)	565 (85%)	9·3 (8·2–10·9)	511 (77%)	134 (20%)	20 (3%)
	Russia (Arkhangelsk) ‡	2000–09	1	60	60 (100%)	1 (2%)	59 (98%)	59 (100%)	59 (100%)	10·7 (9·0–11·8)	51 (86%)	0	8 (14%)
	Slovakia†	2000–07	1	426	423 (99%)	6 (1%)	417 (99%)	284 (68%)	284 (100%)	9·7 (8·6–10·9)	223 (79%)	59 (21%)	2 (1%)
	Slovenia†	1995–2009	1	280	280 (100%)	2 (1%)	278 (99%)	181 (65%)	181 (100%)	13·1 (10·9–15·2)	147 (81%)	33 (18%)	1 (1%)
	Spanish registries	1995–2009	11	1487	1479 (99%)	9 (1%)	1470 (99%)	1152 (78%)	1136 (99%)	10·9 (8·6–13·3)	997 (87%)	123 (11%)	32 (3%)
	Sweden†	1995–2009	1	1162	1162 (100%)	0	1162 (100%)	1145 (99%)	1145 (100%)	11·1 (8·2–13·6)	984 (86%)	154 (13%)	7 (1%)
	Switzerland†	1995–2009	1	853	852 (>99%)	1 (<1%)	851 (>99%)	813 (96%)	812 (>99%)	11·2 (9·2–13·2)	684 (84%)	119 (15%)	10 (1%)
	UK†	1995–2009	3	9868	9797 (99%)	25 (<1%)	9771 (>99%)	6969 (71%)	6881 (99%)	12·5 (10·0–14·9)	5773 (83%)	1129 (16%)	67 (1%)
Oceania		··	··	4218	4158 (99%)	6 (<1%)	4151 (>99%)	3270 (79%)	3143 (96%)	11·2 (9·1–13·9)	2672 (82%)	544 (17%)	54 (2%)
	Australian registries	1995–2009	6	3537	3477 (98%)	2 (<1%)	3474 (>99%)	2607 (75%)	2480 (95%)	9·7 (7·5–12·4)	2145 (82%)	424 (16%)	38 (1%)
	New Zealand†	1995–2009	1	681	681 (100%)	4 (1%)	677 (99%)	663 (98%)	663 (100%)	12·8 (10·7–15·4)	527 (79%)	120 (18%)	16 (2%)
Total		··	198	124 015	122 392 (99%)	565 (<1%)	121 661 (99%)	89 828 (74%)	88 079 (98%)	··	72 474 (81%)	14 573 (16%)	2781 (3%)

Calendar period shows the maximum time span covered by the data. DCO=patients diagnosed from autopsy or Death Certificate Only (% of eligible patients). Microscopic verification=cytology or histology, or morphological verification unspecified (% of patients included in analyses).

* Median follow-up of patients diagnosed during 1995–2004, and IQR.

† National coverage—the data are derived from a population-based cancer registry (registries) covering the entire country.

‡ Korea: Republic of Korea; Russia: Russian Federation.

**Table 2:** 5-year age-standardised net survival in children aged 0–14 years diagnosed with leukaemia

		**Lymphoid leukaemia**						**Acute myeloid leukaemia (ICCC-3 group Ib)**		**Unspecified & other leukaemias (ICCC-3 group Ie)**	
		All lymphoid (Ia)		Precursor cell (Ia1)		Mature B cell (Ia2)					
		n	Net survival (%), 95% CI	n	Net survival (%), 95% CI	n	Net survival (%), 95% CI	n	Net survival (%), 95% CI	n	Net survival (%), 95% CI
**Africa**											
Algerian registries											
	2000–04	19	21·6%*(0·0–45·6)	19	21·6%*(0·0–45·6)	..	..	13	23·9%*(0·0–48·5)	3	..
	2005–09	18	..	17	..	..	..	7	..	2	..
Lesotho†											
	1995–2009	25	43·0%‡(20·9–65·1)	22	39·5%‡(16·3–62·7)	3	..	..	..	2	..
Libya (Benghazi)											
	2003–04	14	70·2% (43·4–96·9)	14	70·2% (43·4–96·9)	..	..	5	..	2	..
Tunisia (Central)											
	1996–99	20	46·1%*(13·2–79·1)	20	46·1%*(13·2–79·1)	..	..	3	..	..	..
	2000–04	6	..	6	..	..	..	2	..	..	..
	2005–07	9	..	9	..	..	..	3	..	..	..
**America (Central and South)**											
Argentina†											
	2000–04	1785	64·6% (62·3–67·0)	1785	64·6% (62·3–67·0)	..	..	..	..	..	..
	2005–09	1711	66·9% (64·4–69·3)	1711	66·9% (64·4–69·3)	..	..	..	..	..	..
Brazilian registries											
	1996–99	67	72·9% (61·8–84·0)	64	71·8% (60·0–83·6)	..	..	13	38·6% (13·8–63·5)	4	..
	2000–04	172	67·9% (60·5–75·3)	168	67·1% (59·6–74·7)	..	..	41	46·1% (32·1–60·1)	16	75·5% (55·0–96·0)
	2005–09	148	66·2% (58·5–73·9)	132	66·4% (58·4–74·3)	10	80·3%‡(48·7–100·0)	25	53·1% (36·9–69·4)	11	55·8% (31·7–79·9)
Chilean registries											
	1998–99	17	41·2% (19·1–63·3)	17	41·2% (19·1–63·3)	..	..	3	..	..	..
	2000–04	21	71·5% (52·7–90·3)	21	71·5% (52·7–90·3)	..	..	2	..	12	100·0%
	2005–08	28	83·9%§(73·3–94·5)	23	77·5% (60·5–94·6)	..	..	9	..	4	..
Colombia (Cali)											
	1995–99	125	40·7% (31·6–49·9)	124	40·4% (31·2–49·6)	..	..	..	..	..	..
	2000–04	137	48·4% (39·4–57·4)	136	48·8% (39·7–57·8)	..	..	..	..	..	..
	2005–09	119	52·4% (42·8–61·9)	117	52·4% (42·8–61·9)	..	..	..	..	..	..
Ecuador (Quito)											
	1995–99	85	64·3% (54·2–74·4)	81	63·7% (53·3–74·1)	..	..	33	49·0%§(35·9–62·1)	5	..
	2000–04	112	63·5% (54·2–72·8)	110	64·1% (54·7–73·6)	..	..	18	50·2% (28·2–72·1)	6	..
	2005–09	98	62·5% (53·5–71·5)	95	63·1% (54·0–72·1)	..	..	16	59·3% (36·8–81·7)	2	..
Puerto Rico†											
	2000–04	79	79·4% (70·5–88·2)	73	79·0% (69·7–88·3)	3	..	17	53·0% (30·3–75·8)	34	72·4%§(60·8–83·9)
	2005–09	84	78·7% (69·5–87·9)	81	78·9% (69·4–88·4)	3	..	18	48·1% (26·4–69·8)	9	..
**America (North)**											
Canada†											
	1995–99	1156	86·0% (83·6–88·4)	1134	85·9% (83·4–88·3)	11	81·9% (60·2–100·0)	235	56·1% (49·5–62·7)	53	68·0% (55·2–80·8)
	2000–04	1092	91·0% (89·0–93·0)	1074	91·0% (89·0–93·0)	10	90·0% (72·4–100·0)	188	64·0% (57·0–70·9)	51	72·7% (60·6–84·9)
	2005–09	1115	90·5% (88·4–92·6)	1097	90·7% (88·6–92·9)	14	80·0% (60·5–99·6)	200	71·8% (65·1–78·5)	62	83·1% (72·9–93·3)
US registries											
	1995–99	7801	82·9% (81·9–83·9)	7670	82·9% (81·9–83·9)	73	77·0% (67·7–86·4)	1574	51·5% (48·9–54·1)	241	68·8% (62·4–75·2)
	2000–04	9025	86·5% (85·7–87·4)	8842	86·6% (85·8–87·5)	128	86·5% (80·1–92·9)	1799	59·7% (57·3–62·1)	285	63·8% (57·7–69·8)
	2005–09	9899	87·7% (86·9–88·5)	9735	87·7% (86·9–88·5)	132	88·7% (83·0–94·5)	1898	63·3% (60·9–65·6)	296	69·8% (64·2–75·4)
**Asia**											
Chinese registries											
	1995–99	29	10·6%§(3·1–18·2)	28	11·1%§(3·3–19·0)	..	..	27	4·2%§(0·0–8·6)	23	8·7% (0·0–18·9)
	2000–04	98	45·6% (36·3–54·9)	84	48·8% (38·8–58·8)	..	..	61	20·9% (10·1–31·7)	69	15·0% (6·7–23·2)
	2005–09	182	69·2% (61·6–76·8)	151	69·4% (61·1–77·8)	10	68·7%‡(40·8–96·6)	61	41·1% (27·8–54·4)	82	26·0% (15·9–36·0)
Cyprus†											
	2004–09	35	83·0%‡ (70·9–95·0)	34	80·8%‡ (68·9–92·6)	1	..	10	60·1%‡(32·2–88·0)	1	..
India (Karunagappally)											
	1995–99	17	59·3% (36·7–81·9)	17	59·3% (36·7–81·9)	..	..	1	..	..	..
	2000–04	14	57·4% (32·6–82·1)	14	57·4% (32·6–82·1)	..	..	4	..	..	..
	2005–09	12	80·2% (60·3–100·0)	12	80·2% (60·3–100·0)	..	..	3	..	2	..
Indonesia (Jakarta)											
	2005–07	14	44·3% (13·4–75·3)	14	44·3% (13·4–75·3)	..	..	6	..	8	..
Israel†											
	1995–99	192	81·4% (74·9–87·8)	188	81·0% (74·5–87·6)	2	..	40	63·2%§(51·6–74·9)	27	74·1% (57·9–90·3)
	2000–04	264	86·3% (81·6–91·1)	258	86·3% (81·5–91·1)	4	..	67	67·5% (56·6–78·4)	27	77·8% (62·5–93·2)
	2005–09	275	84·4% (79·6–89·1)	271	84·5% (79·7–89·3)	1	..	78	66·2% (56·2–76·3)	32	89·2%§(80·7–97·7)
Japanese registries											
	1995–99	294	78·0% (72·9–83·1)	294	78·0% (72·9–83·1)	..	..	122	62·4% (53·7–71·0)	25	60·1% (41·4–78·8)
	2000–04	409	78·0% (73·2–82·9)	406	77·9% (73·1–82·8)	2	..	181	61·2% (53·5–68·9)	24	87·5% (74·6–100·0)
	2005–09	266	82·5% (78·0–86·9)	259	82·2% (77·6–86·8)	4	..	111	69·4% (62·0–76·9)	9	..
Korea†;¶											
	1995–99	1191	63·1% (60·2–66·0)	1171	63·2% (60·3–66·1)	..	..	482	40·1% (35·6–44·5)	167	44·2% (36·8–51·6)
	2000–04	1221	73·0% (70·3–75·6)	1197	73·2% (70·5–75·8)	..	..	504	49·7% (45·4–54·1)	128	55·4% (46·9–63·9)
	2005–09	1171	76·4% (73·9–78·9)	1137	77·1% (74·5–79·6)	43	65·2%‡ (52·9–77·5)	433	53·9% (49·3–58·6)	125	63·9% (55·7–72·1)
Malaysia (Penang)											
	1995–99	51	77·3% (65·5–89·1)	51	77·3% (65·5–89·1)	..	..	22	68·3% (49·3–87·2)	1	..
	2000–04	53	82·8%§(74·2–91·3)	51	81·3%§(72·1–90·4)	..	..	23	74·0% (56·5–91·5)	5	..
	2005–09	59	70·1% (58·4–81·9)	57	69·5% (57·4–81·5)	1	..	22	49·1% (27·8–70·5)	25	76·4% (58·5–94·4)
Mongolia†											
	2005–09	25	18·7% (0·0–40·8)	24	19·5% (0·0–42·5)	..	..	8	..	8	..
Taiwan†											
	1995–99	630	62·8% (58·8–66·9)	601	62·5% (58·4–66·6)	18	72·3% (52·3–92·4)	195	40·8% (33·8–47·8)	52	50·8% (38·2–63·3)
	2000–04	682	72·2% (68·5–76·0)	665	72·0% (68·2–75·8)	15	80·0% (60·5–99·6)	214	48·6% (41·8–55·5)	22	77·3% (60·3–94·4)
	2005–09	623	77·6% (74·1–81·2)	610	78·1% (74·6–81·7)	11	62·1% (39·2–85·0)	205	55·6% (48·8–62·3)	18	63·9% (43·2–84·7)
Thai registries											
	1995–99	102	51·1% (39·4–62·8)	102	51·1% (39·4–62·8)	..	..	18	21·8% (2·1–41·5)	20	52·0% (28·7–75·4)
	2000–04	120	58·9%(49·2–68·5)	120	58·9% (49·2–68·5)	..	..	31	30·5%§(18·0–43·0)	20	37·3% (16·6–58·1)
	2005–09	105	55·0% (45·4–64·7)	102	55·6% (46·0–65·3)	..	..	36	44·7%§(31·8–57·5)	17	57·0% (31·5–82·5)
Turkey (Izmir)											
	1995–99	118	62·8% (53·0–72·7)	116	63·5% (53·7–73·4)	2	..	34	59·2%§(46·1–72·4)	2	..
	2000–04	135	71·0% (62·6–79·3)	131	70·9% (62·5–79·3)	3	..	24	31·2% (13·1–49·4)	1	..
	2005–09	166	73·6% (66·3–81·0)	161	74·2% (66·8–81·7)	4	..	28	50·6%§(35·7–65·4)	8	..
**Europe**											
Austria†											
	1995–99	242	86·8% (81·6–92·0)	240	86·6% (81·3–91·9)	..	..	47	60·1%§(49·1–71·1)	7	..
	2000–04	213	90·1% (85·7–94·5)	208	89·7% (85·2–94·3)	1	..	40	65·0%§(53·4–76·5)	12	66·7% (41·4–92·1)
	2005–09	226	91·1% (86·9–95·3)	221	91·4% (87·2–95·7)	3	..	35	72·6%§(61·2–84·1)	4	..
Belarus†											
	1995–99	286	74·3% (69·1–79·6)	282	74·7% (69·4–79·9)	..	..	..	..	..	..
	2000–04	241	77·5% (72·0–83·0)	235	78·4% (72·9–83·9)	..	..	..	..	..	..
	2005–09	218	88·1% (83·4–92·7)	209	88·3% (83·6–93·0)	17	74·8%‡(54·0–95·7)	..	..	..	..
Belgium†											
	2004–09	396	87·2%‡ (82·9–91·4)	386	86·9%‡ (82·6–91·3)	10	90·0%‡(72·4–100·0)	68	56·6%‡ (43·5–69·6)	2	..
Bulgaria†											
	1995–99	184	57·0% (49·8–64·2)	166	57·6% (50·0–65·2)	..	..	28	27·8%§(15·7–39·9)	37	13·5%§(5·6–21·3)
	2000–04	159	61·8% (53·9–69·6)	154	62·3% (54·3–70·3)	..	..	35	23·1%§(12·7–33·5)	17	29·4% (9·6–49·3)
	2005–09	215	72·0% (65·3–78·8)	215	71·8% (64·9–78·6)	..	..	33	33·3% (18·9–47·7)	6	..
Croatia†											
	1998–99	51	68·7% (56·9–80·6)	44	69·8%§(59·2–80·4)	..	..	13	46·2% (20·7–71·8)	2	..
	2000–04	177	84·1% (77·6–90·6)	160	81·6% (74·2–89·0)	..	..	20	65·0% (44·8–85·3)	4	..
	2005–09	147	85·6% (79·8–91·5)	146	85·7% (79·8–91·7)	17	94·1%‡(83·3–100·0)	28	55·9% (37·6–74·2)	4	..
Denmark†											
	1995–99	166	85·9% (79·7–92·0)	163	85·6% (79·4–91·8)	3	..	32	59·4% (42·9–75·9)	4	..
	2000–04	212	84·2% (78·6–89·8)	209	84·5% (78·9–90·1)	2	..	42	69·1% (55·3–82·9)	10	70·0% (43·3–96·8)
	2005–09	173	87·4% (81·9–93·0)	171	87·2% (81·5–92·9)	2	..	38	68·9% (54·5–83·3)	3	..
Estonia†											
	1995–99	29	53·6%§(40·0–67·3)	29	53·6%§(40·0–67·3)	..	..	7	..	2	..
	2000–04	31	61·0%§(47·8–74·2)	31	61·0%§(47·8–74·2)	..	..	11	36·4% (10·3–62·6)	..	..
	2005–08	13	75·4% (57·0–93·7)	13	75·4% (57·0–93·7)	..	..	6	..	..	..
Finland†											
	1995–99	193	82·3% (76·3–88·4)	193	82·3% (76·3–88·4)	..	..	37	78·4% (65·4–91·5)	4	..
	2000–04	192	84·8% (78·1–91·4)	191	84·7% (78·0–91·4)	..	..	29	65·4%§(52·1–78·8)	12	58·4% (32·0–84·8)
	2005–09	208	82·0% (75·4–88·5)	205	81·8% (75·2–88·4)	2	..	35	69·2% (54·3–84·2)	18	66·9% (45·5–88·4)
France†											
	1995–99	1806	82·4% (80·4–84·3)	1728	82·4% (80·4–84·4)	78	78·7% (69·2–88·2)	380	60·2% (55·1–65·3)	36	52·8% (36·8–68·8)
	2000–04	1883	88·0% (86·3–89·6)	1793	88·1% (86·4–89·8)	89	86·0% (78·7–93·3)	392	62·4% (57·7–67·1)	48	59·8% (46·1–73·5)
	2005–09	1922	89·2% (87·6–90·8)	1828	89·2% (87·6–90·9)	93	88·8% (82·2–95·4)	337	69·4% (64·5–74·2)	71	64·9% (53·0–76·8)
German registries											
	1995–99	481	86·3% (83·0–89·6)	468	86·5% (83·1–89·8)	..	..	107	61·4% (52·3–70·5)	27	74·1% (57·9–90·3)
	2000–04	661	87·3% (84·7–90·0)	642	87·1% (84·4–89·8)	..	..	139	71·0% (63·2–78·8)	16	81·3% (62·8–99·8)
	2005–09	1020	91·6% (89·5–93·6)	989	91·6% (89·5–93·6)	39	93·7%‡ (85·7–100·0)	190	78·2% (72·0–84·3)	20	74·3% (53·0–95·5)
Iceland†											
	1995–99	9	..	9	..	..	..	..	..	1	..
	2000–04	9	..	9	..	..	..	2	..	..	..
	2005–09	11	80·9% (58·1–100·0)	10	90·1% (72·4–100·0)	1	..	4	..	..	..
Ireland†											
	1995–99	149	78·9% (71·5–86·3)	146	79·0% (71·6–86·5)	1	..	29	65·6% (48·7–82·5)	3	..
	2000–04	146	82·9% (76·6–89·2)	145	82·8% (76·5–89·2)	1	..	35	60·0% (44·1–76·0)	7	..
	2005–09	163	84·7% (78·3–91·1)	162	84·7% (78·3–91·1)	1	..	35	65·8% (51·0–80·7)	4	..
Italian registries											
	1995–99	677	83·8% (80·7–86·9)	661	83·7% (80·6–86·9)	..	..	109	60·9% (51·7–70·1)	16	75·1% (54·6–95·5)
	2000–04	740	82·4% (79·1–85·7)	725	82·5% (79·1–85·8)	..	..	124	66·7% (58·5–74·9)	18	77·8% (59·2–96·5)
	2005–09	513	87·9% (85·0–90·9)	507	88·0% (85·0–90·9)	24	76·7%‡(59·0–94·3)	103	68·9% (60·4–77·4)	13	77·2% (55·3–99·2)
Latvia†											
	1995–99	16	50·2% (26·7–73·6)	15	46·8% (22·8–70·9)	..	..	15	40·1% (16·6–63·5)	43	69·6%§(58·4–80·9)
	2000–04	36	91·8% (82·9–100·0)	35	91·6% (82·5–100·0)	..	..	11	45·5% (18·1–72·9)	19	63·3% (42·2–84·3)
	2005–09	45	77·0%§(66·5–87·6)	45	76·5%§(65·8–87·2)	..	..	9	..	2	..
Lithuania†											
	1995–99	103	59·4% (49·3–69·6)	102	59·2% (49·0–69·4)	..	..	12	41·8% (15·9–67·7)	3	..
	2000–04	112	73·7% (64·9–82·5)	106	74·3% (65·3–83·4)	2	..	27	22·3% (7·3–37·2)	..	..
	2005–09	86	68·2% (58·1–78·4)	86	69·0% (58·4–79·6)	..	..	14	44·0% (20·1–67·9)	1	..
Malta†											
	1995–99	19	63·3% (42·3–84·3)	19	63·3% (42·3–84·3)	..	..	..	..	..	..
	2000–04	16	81·4% (62·9–99·8)	16	81·4% (62·9–99·8)	..	..	4	..	..	..
	2005–09	18	83·1% (66·0–100·0)	17	82·5% (64·9–100·0)	..	..	4	..	..	..
Netherlands†											
	1995–99	529	81·1% (77·2–84·9)	527	81·0% (77·1–84·9)	2	..	92	58·6% (48·0–69·2)	12	75·1% (51·8–98·5)
	2000–04	586	84·0% (80·6–87·4)	582	84·0% (80·6–87·4)	1	..	109	53·7% (43·7–63·8)	11	81·9% (60·2–100·0)
	2005–09	537	86·2% (82·9–89·6)	533	86·2% (82·9–89·6)	4	..	106	58·6% (48·2–69·0)	6	..
Norway†											
	1995–99	180	79·2% (71·6–86·9)	177	79·1% (71·4–86·8)	2	..	42	54·3% (39·6–69·0)	3	..
	2000–04	182	87·7% (82·5–93·0)	178	87·7% (82·3–93·1)	4	..	37	65·5%§(53·1–77·9)	2	..
	2005–09	169	89·7% (84·5–94·9)	169	89·7% (84·5–94·9)	..	..	29	67·3% (50·1–84·5)	2	..
Poland (Wroclaw)											
	2000–04	33	68·9%§(56·6–81·2)	33	68·9%§(56·6–81·2)	..	..	8	..	..	..
	2005–09	63	80·9% (70·8–90·9)	62	80·9% (70·8–90·9)	1	..	16	66·7% (43·6–89·9)	..	..
Portugal†											
	1998–99	45	66·0%§(54·3–77·6)	45	66·0%§(54·3–77·6)	..	..	17	53·0% (30·2–75·8)	1	..
	2000–04	257	79·3% (73·8–84·8)	238	79·2% (73·5–84·8)	..	..	65	53·3% (41·7–65·0)	7	..
	2005–09	209	84·0% (78·8–89·2)	191	83·7% (78·1–89·2)	21	94·5%‡(84·2–100·0)	52	60·4% (48·6–72·3)	12	69·7% (42·5–96·8)
Russia (Arkhangelsk)¶											
	2000–04	27	55·7% (37·3–74·0)	23	52·2% (32·4–72·1)	1	..	..	..	6	..
	2005–09	24	74·8% (57·7–92·0)	23	73·1% (55·0–91·2)	..	..	..	..	2	..
Slovakia†											
	2000–04	132	79·4% (72·1–86·7)	131	79·3% (72·0–86·6)	..	..	35	45·0% (30·0–59·9)	2	..
	2005–07	91	79·1% (70·4–87·7)	89	78·3% (69·5–87·2)	..	..	24	46·7% (23·2–70·3)	..	..
Slovenia†											
	1995–99	50	83·4%§(74·4–92·3)	50	83·4%§(74·4–92·3)	..	..	7	..	..	..
	2000–04	49	89·7%§(81·8–97·6)	49	89·7%§(81·8–97·6)	..	..	11	63·7% (36·8–90·5)	..	..
	2005–09	48	75·8%§(65·4–86·2)	43	81·3% (69·7–92·9)	5	..	15	79·5% (59·5–99·5)	1	..
Spanish registries											
	1995–99	296	74·4% (68·9–79·9)	290	74·1% (68·5–79·7)	..	..	38	47·3%§(34·7–59·9)	17	58·9% (36·4–81·4)
	2000–04	319	81·6% (77·0–86·1)	308	81·9% (77·3–86·5)	..	..	39	64·0% (50·0–78·0)	10	70·0% (43·3–96·8)
	2005–09	382	83·7% (79·5–87·9)	362	84·2% (80·0–88·4)	30	80·0%‡ (67·4–92·7)	46	60·2% (47·2–73·2)	5	..
Sweden†											
	1995–99	368	84·2% (79·8–88·7)	344	85·0% (80·5–89·5)	2	..	50	72·2%§(61·6–82·7)	..	..
	2000–04	333	85·9% (81·7–90·0)	313	86·8% (82·6–90·9)	..	..	56	56·3%§(45·8–66·8)	3	..
	2005–09	283	84·5% (80·1–88·9)	230	85·5% (80·9–90·1)	2	..	48	65·9%§(55·5–76·4)	4	..
Switzerland†											
	1995–99	224	86·0% (80·9–91·1)	220	85·6% (80·3–90·8)	..	..	42	53·4% (38·7–68·1)	..	..
	2000–04	229	87·6% (82·8–92·4)	222	87·2% (82·3–92·1)	..	..	43	53·7%§(42·3–65·2)	6	..
	2005–09	231	87·9% (83·3–92·6)	225	88·3% (83·7–92·9)	14	100·0%‡(100·0–100·0)	34	75·2%§(64·0–86·3)	4	..
UK†											
	1995–99	1896	79·1% (77·0–81·2)	1871	79·2% (77·0–81·3)	..	..	371	58·6% (53·4–63·8)	27	70·5% (53·6–87·3)
	2000–04	1976	85·9% (84·2–87·7)	1954	86·0% (84·3–87·8)	..	..	390	65·6% (60·7–70·5)	18	55·6% (33·7–77·5)
	2005–09	1901	89·3% (87·7–90·9)	1893	89·2% (87·6–90·8)	51	77·8%‡ (66·4–89·1)	368	68·1% (63·2–73·1)	22	76·3% (58·4–94·1)
**Oceania**											
Australian registries											
	1995–99	685	82·8% (79·6–86·0)	682	82·9% (79·7–86·1)	..	..	125	53·4% (44·6–62·2)	13	46·2% (20·7–71·7)
	2000–04	833	86·0% (83·3–88·7)	825	86·2% (83·4–88·9)	..	..	170	70·8% (63·8–77·8)	16	68·9% (47·0–90·8)
	2005–09	627	88·8% (86·0–91·6)	617	89·0% (86·2–91·8)	17	82·0%‡(64·0–99·9)	129	68·5% (60·3–76·6)	9	..
New Zealand†											
	1995–99	168	82·8% (76·4–89·3)	167	82·6% (76·0–89·1)	1	..	42	67·6%§(56·3–78·9)	5	..
	2000–04	183	85·2% (79·3–91·2)	182	85·8% (79·9–91·7)	1	..	44	68·5%§(57·7–79·4)	6	..
	2005–09	176	89·3% (83·8–94·8)	176	89·3% (83·8–94·8)	..	..	34	74·9%§(63·7–86·1)	5	..

Data stratified by continent, country, and calendar period of diagnosis (1995–99, 2000–04, and 2005–09). Underlined estimates are not age-standardised. ICCC-3= International Classification of Childhood Cancer, 3rd edition.

* Estimate judged as less reliable.

† National coverage—the data are derived from a population-based cancer registry (registries) covering the entire country.

‡ Estimate based on merging data for 2 (or all 3) calendar periods.

§ Age-standardised estimate computed by pooling two age-specific estimates and re-estimation.

¶ Korea: Republic of Korea; Russia: Russian Federation.
