# Correction to *Lancet Haematol* 2017; 4: e202–17

**DOI:** 10.1016/S2352-3026(17)30067-4

**Published:** 2017-04-28

*Bonaventure A, Harewood R, Stiller CA, et al. Worldwide comparison of survival from childhood leukaemia for 1995–2009, by subtype, age, and sex (CONCORD-2): a population-based study of individual data for 89 828 children from 198 registries in 53 countries.* Lancet Haematol *2017; **4:** e202–17—*The appendix for this article (pp 2, 3) has been corrected. The list of CONCORD Working Group members for the Registro de Câncer de São Paulo (Brazil) should only include MRDO Latorre and LF Tanaka. The correct affiliation for N Bhoo-Pathy is University of Malaya (Malaysia), and the correct affiliation for O Chimedsuren is Mongolian National University of Medical Sciences–MNUMS (Mongolia). This correction has been made to the online version as of April 28, 2017.

