# Supplementary material for: Worldwide comparison of survival from childhood leukaemia for 1995–2009, by subtype, age, and sex (CONCORD-2): a population-based study of individual data for 89 828 children from 198 registries in 53 countries
Source: Lancet Haematol. 2017 Apr 11;4(5):e202–17. doi: 10.1016/S2352-3026(17)30052-2 (PMC5418564; doi:10.1016/S2352-3026(17)30052-2)
Supplement: Supplementary appendix [file mmc1.pdf]

THE LANCET
Haematology

Supplementary appendix

This appendix formed part of the original submission and has been peer reviewed. We post it as supplied by the authors.

Supplement to: Bonaventure A, Harewood R, Stiller CA, et al, and the CONCORD Working Group. Worldwide comparison of survival from childhood leukaemia for 1995–2009, by subtype, age, and sex (CONCORD-2): a population-based study of data including 89 828 children from 198 registries in 53 countries. *Lancet Haematol* 2017; published online April 11. http://dx.doi.org/10.1016/S2352-3026(17)30052-2.

This supplementary appendix has been corrected. The corrected version first appeared at thelancet.com/haematology on April 28, 2017.

Supplementary appendix to:

Worldwide comparison of survival from childhood leukaemia 1995-2009, by subtype, age, and sex (CONCORD-2): a population-based study of individual data for 89 828 children from 198 registries in 53 countries

Bonaventure A, Harewood R, Stiller CA, Gatta G, Clavel J, Stefan DC, Carreira H, Spika D, Marcos-Gragera R, Peris-Bonet R, Piñeros M, Sant M, Kuehni CE, Murphy MFG, Coleman MP, Allemani C, and the CONCORD Working Group

**CONCORD Working Group**	p2
**Web-table 1**: Definition of leukaemia sub-types, based on morphology codes from the International Classification of Diseases for Oncology, third edition (ICD-O-3)[1], with groupings from the International Classification of Childhood Cancer, third edition[2]	p5
**Web-table 2**: Precursor-cell lymphoid leukaemia (ICCC-3 Ia1) - Five-year net survival (NS, %) by age group and sex: continent, country and calendar period of diagnosis	p6
**Web-table 3**: Acute myeloid leukaemia (ICCC-3 Ib) - Five-year net survival (NS, %) by age group and sex: continent, country and calendar period of diagnosis	p15

CONCORD Working Group

Africa—***Algeria***: S Bouzbid (Registre du Cancer d'Annaba); M Hamdi-Chérif*, Z Zaidi (Registre du Cancer de Sétif); ***Gambia***: E Bah, R Swaminathan (National Cancer Registry); ***Lesotho***: SH Nortje (Children's Haematology Oncology Clinics - Lesotho); ***Libya***: MM El Mistiri (Benghazi Cancer Registry); ***Mali***: S Bayo, B Malle (Kankou Moussa University); ***Mauritius***: SS Manraj, R Sewpaul-Sungkur (Mauritius Cancer Registry); ***Nigeria***: A Fabowale, OJ Ogunbiyi* (Ibadan Cancer Registry); ***South Africa***: D Bradshaw, NIM Somdyala (Eastern Cape Province Cancer Registry); DC Stefan (Umtata University); ***Sudan***: M Abdel-Rahman (University of Khartoum); ***Tunisia***: L Jaidane, M Mokni (Registre du Cancer du Centre Tunisien).

America (Central and South)—***Argentina***: I Kumcher, F Moreno (National Childhood Cancer Registry – National Cancer Institute); MS González, EA Laura (Registro Regional de Tumores del Sur de la Provincia de Buenos Aires); SB Espinola, GH Calabrano (Registro Poblacional de Tumores de la Provincia del Chubut); B Carballo Quintero, R Fita (Registro Provincial de Tumores de Córdoba); DA Garcilazo, PL Giacciani (Entre Rios Cancer Registry); MC Diumenjo, WD Laspada (Registro Provincial de Tumores de Mendoza); MA Green, MF Lanza (Registro de Cáncer de Santa Fe); SG Ibañez (Cancer Registry of Tierra del Fuego Province); ***Brazil***: CA Lima, E Lobo de Oliveira (Registro de Câncer de Base Populacional de Aracaju); C Daniel, C Scandiuzzi (Cancer Registry of Distrito Federal); PCF De Souza, CD Melo (Registro de Câncer de Base Populacional de Cuiabá); K Del Pino, C Laporte (Registro de Curitiba); MP Curado, JC de Oliveira (Registro de Goiânia); CLA Veneziano, DB Veneziano (Registro de Câncer de Base Populacional de Jahu); MRDO Latorre, LF Tanaka (Registro de Câncer de São Paulo); G Azevedo e Silva* (University of Rio de Janeiro); ***Chile***: JC Galaz, JA Moya (Registro Poblacional de Cáncer Region de Antofagasta); DA Herrmann, S Vargas (Registro Poblacional Region de Los Rios); ***Colombia***: VM Herrera, CJ Uribe (Registro Poblacional de Cáncer Area Metropolitana de Bucaramanga); LE Bravo (Cali Cancer Registry); NE Arias-Ortiz (Registro Poblacional de Cáncer de Manizales); DM Jurado, MC Yépez (Registro Poblacional de Cáncer del Municipio de Pasto); ***Cuba***: YH Galán, P Torres (Registro Nacional de Cáncer de Cuba); ***Ecuador***: F Martínez-Reyes, ML Pérez-Meza (Cuenca Tumor Registry); L Jaramillo, R Quinto (Guayaquil Cancer Registry); P Cueva, JG Yépez (Quito Cancer Registry); ***Puerto Rico***: CR Torres-Cintrón, G Tortolero-Luna (Puerto Rico Central Cancer Registry); ***Uruguay***: R Alonso, E Barrios (Registro Nacional de Cáncer).

America (North)—***Canada***: C Nikiforuk, L Shack (Alberta Cancer Registry); AJ Coldman, RR Woods (British Columbia Cancer Registry); G Noonan, D Turner* (Manitoba Cancer Registry); E Kumar, B Zhang (New Brunswick Provincial Cancer Registry); FR McCrate, S Ryan (Newfoundland and Labrador Cancer Registry); H Hannah (Northwest Territories Cancer Registry); RAD Dewar, M MacIntyre (Nova Scotia Surveillance and Epidemiology Unit); A Lalany, M Ruta (Nunavut Department of Health and Social Services); L Marrett, DE Nishri* (Ontario Cancer Registry); C McClure, KA Vriends (Prince Edward Island Cancer Registry); C Bertrand, R Louchini (Registre Québécois du Cancer); KI Robb, H Stuart-Panko (Saskatchewan Cancer Registry); S Demers, S Wright (Yukon Government); ***USA***: JT George, X Shen (Alabama Statewide Cancer Registry); JT Brockhouse, DK O'Brien (Alaska Cancer Registry); KC Ward (Georgia Comprehensive Cancer Registry; Metropolitan Atlanta Registry); L Almon (Metropolitan Atlanta Registry); J Bates (California State Cancer Registry); R Rycroft (Colorado Central Cancer Registry); L Mueller, C Phillips (Connecticut Tumor Registry); H Brown, B Cromartie (Delaware Cancer Registry); AG Schwartz, F Vigneau (Metropolitan Detroit Cancer Surveillance System); JA MacKinnon, B Wohler (Florida Cancer Data System); AR Bayakly (Georgia Comprehensive Cancer Registry); CA Clarke, SL Glaser (Greater Bay Area Cancer Registry); D West (Cancer Registry of Greater California); MD Green, BY Hernandez (Hawaii Tumor Registry); CJ Johnson, D Jozwik (Cancer Data Registry of Idaho); ME Charlton, CF Lynch (State Health Registry of Iowa); B Huang, TC Tucker* (Kentucky Cancer Registry); D Deapen, L Liu (Los Angeles Cancer Surveillance Program); MC Hsieh, XC Wu (Louisiana Tumor Registry); K Stern (Maryland Cancer Registry); ST Gershman, RC Knowlton (Massachusetts Cancer Registry); J Alverson, GE Copeland (Michigan State Cancer Surveillance Program); DB Rogers (Mississippi Cancer Registry); D Lemons, LL Williamson (Montana Central Tumor Registry); M Hood (Nebraska Cancer Registry); GM Hosain, JR Rees (New Hampshire State Cancer Registry); KS Pawlish, A Stroup (New Jersey State Cancer Registry); C Key, C Wiggins (New Mexico Tumor Registry); AR Kahn, MJ Schymura (New York State Cancer Registry); G Leung, C Rao (North Carolina Central Cancer Registry); L Giljahn, B Warther (Ohio Cancer Incidence Surveillance System); A Pate (Oklahoma Central Cancer Registry); M Patil, SS Schubert (Oregon State Cancer Registry); JJ Rubertone, SJ Slack (Pennsylvania Cancer Registry); JP Fulton, DL Rousseau (Rhode Island Cancer Registry); TA Janes, SM Schwartz (Seattle Cancer Surveillance System); SW Bolick, DM Hurley (South Carolina Central Cancer Registry); J Richards, MA Whiteside (Tennessee Cancer Registry); LM Nogueira (Texas Cancer Registry); K Herget, C Sweeney (Utah Cancer Registry); J Martin, S Wang (Virginia Cancer Registry); DG Harrelson, MB Keitheri Cheteri (Washington State Cancer Registry); S Farley, AG Hudson (West Virginia Cancer Registry); R Borchers, L Stephenson (Wisconsin Department of Health Services); JR

Espinoza (Wyoming Cancer Surveillance Program); HK Weir* (Centers for Disease Control and Prevention); BK Edwards* (National Cancer Institute).

Asia—***China***: N Wang, L Yang (Beijing Cancer Registry); JS Chen (Changle City Cancer Registry); GH Song (Cixian Cancer Registry); XP Gu (Dafeng County Center for Disease Control and Prevention); P Zhang (Dalian Centers for Disease Prevention and Control); HM Ge (Donghai County Center for Disease Prevention and Control); DL Zhao (Feicheng County); JH Zhang (Ganyu Center for Disease Prevention and Control); FD Zhu (Guanyun Cancer Registry); JG Tang (Haimen Cancer Registry); Y Shen (Haining City Cancer Registry); J Wang (Jianhu Cancer Registry); QL Li (Jiashan County Cancer Registry); XP Yang (Jintan Cancer Registry); J Dong, W Li (Lianyungang Center for Disease Prevention and Control); LP Cheng (Henan Province Central Cancer Registry); JG Chen (Qidong County Cancer Registry); QH Huang (Sihui Cancer Registry); SQ Huang (Taixing Cancer Registry); GP Guo (Cancer Institute of Yangzhong City); K Wei (Zhongshan City Cancer Registry); WQ Chen*, H Zeng (National Central Cancer Registry China); ***Cyprus***: AV Demetriou, P Pavlou (Cyprus Cancer Registry); ***Hong Kong***: WK Mang, KC Ngan (Hong Kong Cancer Registry); ***India***: R Swaminathan (Chennai Cancer Registry); AC Kataki, M Krishnatreya (Guwahati Cancer Registry); PA Jayalekshmi, P Sebastian (Karunagappally Cancer Registry); SD Sapkota, Y Verma (Population Based Cancer Registry, Sikkim); A Nandakumar* (National Centre for Disease Informatics and Research; National Cancer Registry Programme); ***Indonesia***: E Suzanna (Jakarta Cancer Registry); ***Israel***: L Keinan-Boker, BG Silverman (Israel National Cancer Registry); ***Japan***: H Ito, H Nakagawa (Aichi Cancer Registry); M Hattori, Y Kaizaki (Fukui Cancer Registry); H Sugiyama, M Utada (Hiroshima Prefecture Cancer Registry); K Katayama, H Narimatsu (Kanagawa Cancer Registry); S Kanemura (Miyagi Prefectural Cancer Registry); T Koike (Niigata Prefecture Cancer Registry); I Miyashiro (Osaka Cancer Registry); M Yoshii (Saga Prefectural Cancer Registry); I Oki (Tochigi Prefectural Cancer Registry); A Shibata (Yamagata Cancer Registry); T Matsuda* (National Cancer Center); ***Jordan***: O Nimri (Jordan National Cancer Registry); ***Malaysia***: A Ab Manan (Penang Cancer Registry); N Bhoo-Pathy (University of Malaya); ***Mongolia***: S Tuvshingerel (Cancer Registry of Mongolia); O Chimedsuren (Mongolian National University of Medical Sciences – MNUMS); ***Qatar***: AHM Al Khater, MM El Mistiri (Qatar Cancer Registry); ***Saudi Arabia***: H Al-Eid (Saudi National Cancer Registry); ***South Korea***: KW Jung, YJ Won (Korea Central Cancer Registry); ***Taiwan***: CJ Chiang, MS Lai (Taiwan Cancer Registry); ***Thailand***: K Suwanrungruang, S Wiangnon (Khon Kaen Provincial Registry); K Daoprasert, D Pongnikorn (Lampang Cancer Registry); SL Geater, H Sriplung (Songkhla Cancer Registry); ***Turkey***: S Eser, CI Yakut (Izmir Cancer Registry).

Europe—***Austria***: M Hackl (Austrian National Cancer Registry); H Mühlböck, W Oberaigner (Tyrol Cancer Registry); ***Belarus***: AA Zborovskaya (Belarus Childhood Cancer Subregistry); OV Aleinikova (Belarusian Research Center for Pediatric Oncology, Hematology and Immunology); ***Belgium***: K Henau, L Van Eycken (Belgian Cancer Registry); ***Bulgaria***: N Dimitrova, Z Valerianova (Bulgarian National Cancer Registry); ***Croatia***: M Šekerija (Croatian National Cancer Registry); ***Czech Republic***: M Zvolský (Czech National Cancer Registry); ***Denmark***: G Engholm, H Storm* (Danish Cancer Society); ***Estonia***: K Innos, M Mägi (Estonian Cancer Registry); ***Finland***: N Malila, K Seppä (Cancer Society of Finland); ***France***: J Jégu, M Velten (Bas-Rhin General Cancer Registry); E Cornet, X Troussard (Registre Régional des Hémopathies Malignes de Basse Normandie); AM Bouvier, J Faivre (Burgundy Digestive Cancer Registry); AV Guizard (Calvados General Cancer Registry); V Bouvier, G Launoy (Calvados Digestive Cancer Registry); P Arveux (Côte-d'Or Gynaecologic Cancer Registry); M Maynadié, M Mounier (Côte-d'Or Haematopoietic Malignancies Registry); E Fournier, AS Woronoff (Doubs and Belfort Territory General Cancer Registry); M Daoulas (Finistère Cancer Registry); J Clavel (National Registry of Childhood Haematopoietic Malignancies); S Le Guyader-Peyrou, A Monnereau (Gironde Haematopoietic Malignancies Registry); B Trétarre (Hérault General Cancer Registry); M Colonna (Isère General Cancer Registry); A Cowppli-Bony, F Molinié (Loire-Atlantique-Vendée Cancer Registry); S Bara, D Degré (Manche General Cancer Registry); O Ganry, B Lapôtre-Ledoux (Somme General Cancer Registry); P Grosclaude (Tarn General Cancer Registry); J Estève (Hospices Civils de Lyon); F Bray*, M Piñeros* (International Agency for Research on Cancer); F Sassi (Organisation for Economic Co-operation and Development); ***Germany***: R Stabenow (Common Cancer Registry of the Federal States); A Eberle (Bremen Cancer Registry); C Erb, A Nennecke (Hamburg Cancer Registry); J Kieschke, E Sirri (Epidemiological Cancer Registry of Lower Saxony); H Kajueter (North Rhine Westphalia Cancer Registry); K Emrich, SR Zeissig (Rhineland Palatinate Cancer Registry); B Holleczek (Saarland Cancer Registry); N Eisemann, A Katalinic (Schleswig-Holstein Cancer Registry); H Brenner (German Cancer Research Center); ***Gibraltar***: RA Asquez, V Kumar (Gibraltar Cancer Registry); ***Iceland***: EJ Ólafsdóttir, L Tryggvadóttir (Icelandic Cancer Registry); ***Ireland***: H Comber, PM Walsh (National Cancer Registry); H Sundseth* (European Institute of Women's Health); ***Italy***: E Devigili, G Mazzoleni (Registro Tumori Alto Adige); A Giacomin (Registro Tumori Biella); F Bella, M Castaing (Integrated Cancer Registry of Catania-Messina-Siracusa-Enna); A Sutera (Registro Tumori Catanzaro); G Gola (Registro Tumori della Provincia di Como); S Ferretti (Registro Tumori della Provincia di Ferrara); D Serraino, A Zucchetto (Registro Tumori del Friuli Venezia Giulia); R Lillini, M Vercelli (Registro Tumori Regione Liguria); S Busco, F Pannozzo (Registro Tumori della Provincia di Latina); S Vitarelli (Registro Tumori

della Provincia di Macerata); P Ricci (Registro Tumori Mantova); C Pascucci (Registro Tumori Marche Childhood); M Autelitano (Registro Tumori Milano); C Cirilli, M Federico (Registro Tumori della Provincia di Modena); M Fusco, MF Vitale (Registro Tumori della ASL Napoli 3 sud); M Usala (Nuoro Cancer Registry); R Cusimano, W Mazzucco (Registro Tumori di Palermo e Provincia); M Michiara, P Sgargi (Registro Tumori della Provincia di Parma); MM Maule, C Sacerdote (Piedmont Childhood Cancer Registry); R Tumino (Registro Tumori della Provincia di Ragusa); E Di Felice, M Vicentini (Registro Tumori Reggio Emilia); F Falcini (Registro Tumori della Romagna); L Cremone (Registro Tumori Salerno); M Budroni, R Cesaraccio (Registro Tumori della Provincia di Sassari); ML Contrino, F Tisano (Registro Tumori Siracusa); AC Fanetti, S Maspero (Registro Tumori della Provincia di Sondrio); G Candela, T Scuderi (Registro Tumori Trapani); MA Gentilini, S Piffer (Registro Tumori Trento); S Rosso, L Sacchetto (Registro Tumori Piemonte Città di Torino); A Caldarella (Registro Tumori della Regione Toscana); F La Rosa, F Stracci (Registro Tumori Umbro di Popolazione); P Contiero, G Tagliabue (Registro Tumori Lombardia, Provincia di Varese); AP Dei Tos, M Zorzi (Registro Tumori Veneto); R Zanetti* (International Association of Cancer Registries); P Baili, F Berrino*, G Gatta, M Sant* (Fondazione IRCCS Istituto Nazionale dei Tumori); R Capocaccia*, R De Angelis (National Centre for Epidemiology); ***Latvia***: E Liepina, A Maurina (Latvian Cancer Registry); ***Lithuania***: G Smailyte (Lithuanian Cancer Registry); ***Malta***: D Agius, N Calleja (Malta National Cancer Registry); ***Netherlands***: S Siesling, O Visser (Comprehensive Cancer Centre of the Netherlands); ***Norway***: S Larønningen, B Møller (The Cancer Registry of Norway); ***Poland***: A Dyzmann-Sroka, M Trojanowski (Greater Poland Cancer Registry); S Góźdź, R Mężyk (Cancer Registry of Kielce); M Grądalska-Lampart, AU Radziszewska (Podkarpackie Cancer Registry); JA Didkowska, U Wojciechowska (National Cancer Registry); J Błaszczyk, K Kępska (Lower Silesian Cancer Registry); M Bielska-Lasota, K Kwiatkowska (National Institute of Public Health - NIH); ***Portugal***: G Forjaz, RA Rego (Registo Oncológico Regional dos Açores); J Bastos, MA Silva (Registo Oncológico Regional do Centro); L Antunes, MJ Bento (Registo Oncológico Regional do Norte); A Mayer-da-Silva, A Miranda (Registo Oncólogico Regional do Sul); ***Romania***: D Coza, AI Todescu (Cancer Institute I. Chiricuta); ***Russian Federation***: MY Valkov (Arkhangelsk Regional Cancer Registry); ***Slovakia***: J Adamcik, C Safaei Diba (National Cancer Registry of Slovakia); ***Slovenia***: M Primic-Žakelj, T Žagar (Cancer Registry of Republic of Slovenia); J Stare (University of Ljubljana); ***Spain***: E Almar, A Mateos (Registro de Cáncer de Albacete); JR Quirós (Registro de Tumores del Principado de Asturias); J Bidaurrazaga, N Larrañaga (Basque Country Cancer Registry); JM Díaz García, AI Marcos (Registro de Cáncer de Cuenca); R Marcos-Gragera, ML Vilardell Gil (Registre de Càncer de Girona); E Molina, MJ Sánchez (Registro de Cáncer de Granada); P Franch Sureda, M Ramos Montserrat (Mallorca Cancer Registry); MD Chirlaque, C Navarro (Murcia Cancer Registry); EE Ardanaz, CC Moreno-Iribas (Registro de Cáncer de Navarra); R Fernández-Delgado, R Peris-Bonet (Registro Español de Tumores Infantiles (RETI-SEHOP)); J Galceran (Tarragona Cancer Registry); ***Sweden***: S Khan, M Lambe (Swedish Cancer Registry); ***Switzerland***: B Camey (Registre Fribourgeois des Tumeurs); C Bouchardy, M Usel (Geneva Cancer Registry); SM Ess (Cancer Registry Grisons and Glarus); C Herrmann (Cancer Registry Grisons and Glarus; Cancer Registry of St Gallen-Appenzell); JL Bulliard, M Maspoli-Conconi (Registre Neuchâtelois des Tumeurs); H Frick (Cancer Registry of St Gallen-Appenzell); CE Kuehni, M Schindler (Swiss Childhood Cancer Registry); A Bordoni, A Spitale (Registro Tumori Cantone Ticino); A Chiolero, I Konzelmann (Registre Valaisan des Tumeurs); SI Dehler, KL Matthes (Krebsregister der Kantone Zürich und Zug); ***United Kingdom***: J Rashbass, CA Stiller* (Public Health England); D Fitzpatrick, A Gavin (Northern Ireland Cancer Registry); F Bannon (Queens University, Belfast); RJ Black, DH Brewster (Scottish Cancer Registry); DW Huws, C White (Welsh Cancer Intelligence & Surveillance Unit); P Finan (Leeds General Infirmary); C Allemani*, A Bonaventure, H Carreira, MP Coleman*, V Di Carlo, R Harewood, K Liu, M Matz, L Montel, M Nikšić, B Rachet*, N Sanz, D Spika (London School of Hygiene & Tropical Medicine); R Stephens* (National Cancer Research Institute, London); M Peake (University of Leicester), MFG Murphy (University of Oxford).

Oceania—***Australia***: E Chalker, L Newman (Australian Capital Territory Cancer Registry); D Baker, MJ Soeberg (NSW Cancer Registry); J Aitken, C Scott (Queensland Cancer Registry); BC Stokes, A Venn (Tasmanian Cancer Registry); H Farrugia, GG Giles (Victorian Cancer Registry); T Threlfall (Western Australian Cancer Registry); D Currow*, H You (Cancer Institute NSW); ***New Zealand***: J Hendrix, C Lewis (New Zealand Cancer Registry).

*CONCORD Steering Committee

Web-table 1

Definition of leukaemia sub-types, based on morphology codes from the International Classification of Diseases for Oncology, third edition (ICD-O-3)[1], with groupings from the International Classification of Childhood Cancer, third edition[2]

ICD-O-3 morphology code	Label (main title)	ICCC-3 subgroup (if any)
**Lymphoid leukaemias (group Ia)**		
9835	Precursor cell lymphoblastic leukaemia, NOS	Precursor-cell
9836	Precursor B-cell lymphoblastic leukaemia	Precursor-cell
9837	Precursor T-cell lymphoblastic leukaemia	Precursor-cell
9823	B-cell chronic lymphocytic leukaemia/small lymphocytic lymphoma	Mature B-cell
9826	Burkitt cell leukaemia	Mature B-cell
9832	Prolymphocytic leukaemia, NOS	Mature B-cell
9833	Prolymphocytic leukaemia, B-cell type	Mature B-cell
9940	Hairy cell leukaemia	Mature B-cell
9827	Adult T-cell leukaemia/lymphoma (HTLV-1 positive)	Mature T-cell and NK cell
9831	T-cell large granular lymphocytic leukaemia	Mature T-cell and NK cell
9834	Prolymphocytic leukaemia, T-cell type	Mature T-cell and NK cell
9948	Aggressive NK-cell leukaemia	Mature T-cell and NK cell
9820	Lymphoid leukaemia, NOS	Lymphoid leukaemia, NOS
**Acute myeloid leukaemias (group Ib)**		
9840	Acute myeloid leukaemia, M6 type	
9861	Acute myeloid leukaemia, NOS	
9866	Acute promyelocytic leukaemia, t(15;17) (q22;q11-12)	
9867	Acute myelomonocytic leukaemia	
9870	Acute basophilic leukaemia	
9871	Acute myeloid leukaemia with abnormal marrow eosinophils	
9872	Acute myeloid leukaemia, minimal differentiation	
9873	Acute myeloid leukaemia without maturation	
9874	Acute myeloid leukaemia with maturation	
9891	Acute monocytic leukaemia	
9895	Acute myeloid leukaemia with multilineage dysplasia	
9896	Acute myeloid leukaemia, t(8;21)(q22;q22)	
9897	Acute myeloid leukaemia, 11q23 abnormalities	
9910	Acute megakaryoblastic leukaemia	
9920	Therapy-related acute myeloid leukaemia, NOS	
9931	Acute panmyelosis with myelofibrosis	
**Unspecified and other specified leukaemias (group Ie)**		
9800	Leukaemia, NOS	
9801	Acute leukaemia, NOS	
9805	Acute biphenotypic leukaemia	
9860	Myeloid leukaemia, NOS	
9930	Myeloid sarcoma	

References

1. Fritz AG, Percy C, Jack A, Shanmugaratnam K, Sobin LH, Parkin DM, Whelan SL, editors. International Classification of Diseases for Oncology (ICD-O). 3rd ed. Geneva: World Health Organization; 2000.
2. Steliarova-Foucher E, Stiller C, Lacour B, Kaatsch P. International Classification of Childhood Cancer, third edition. *Cancer* 2005; **103**: 1457-67.

Web-table 2

Precursor-cell lymphoid leukaemia (ICCC-3 Ia1) - Five-year net survival (NS, %) by age group and sex: continent, country and calendar period of diagnosis

Net survival estimates by sex were age-standardised where possible. Estimates in italics are not age-standardised

* National coverage – the data are derived from a population-based cancer registry (registries) covering the entire country

[M] Estimate based on merging data for 2 (or all 3) calendar periods

[R] Age-standardised estimate computed by pooling two age-specific estimates and re-estimation

[§] Estimate judged as less reliable

‡ Korea: Republic of Korea; Russia: Russian Federation

Web-table 2: Precursor-cell lymphoid leukaemia (ICCC3 Ia1) - Five-year net survival (NS, %) by age group and sex: continent, country and calendar period of diagnosis

Calendar	Age group (years)												Sex					
	<1			1-4			5-9			10-14			Boys			Girls		
	N	NS %	95% CI	N	NS %	95% CI	N	NS %	95% CI	N	NS %	95% CI	N	NS %	95% CI	N	NS %	95% CI
**AFRICA**																		
Algerian registries																		
2000-2004	1			3			6			9			11	*56·5* [§]	*25·6 - 87·3*	8		
2005-2009				7			5			5			9			8		
Lesotho *																		
1995-2009				5			13	**25·3** [M]	0·7 - 49·9	4			13	*43·6* [M]	*14·8 - 72·3*	9		
Libya (Benghazi)																		
2003-2004				8			4			2			7			7		
Tunisia (Central)																		
1996-1999				9			7			4			15	*53·4* [§]	*16·2 - 90·7*	5		
2000-2004				2			3			1			3			3		
2005-2007				2			2			5			8			1		
**AMERICA (Central and South)**																		
Argentina *																		
2000-2004	68	**32·0**	20·7 - 43·3	768	**72·9**	69·6 - 76·1	560	**66·6**	62·5 - 70·6	389	**57·8**	52·8 - 62·8	952	**63·5**	60·2 - 66·8	833	**65·6**	62·1 - 69·1
2005-2009	58	**37·0**	23·9 - 50·0	768	**76·9**	73·7 - 80·0	472	**69·5**	65·3 - 73·8	413	**57·0**	52·0 - 62·0	961	**66·3**	63·0 - 69·7	750	**67·2**	63·6 - 70·8
Brazilian registries																		
1996-1999	3			29	**79·5**	65·1 - 94·0	21	**76·2**	58·5 - 94·0	11	**63·7**	36·9 - 90·6	32	**75·5** [R]	64·8 - 86·2	32	*72·1*	*56·7 - 87·5*
2000-2004	6			76	**83·2**	74·7 - 91·6	51	**68·7**	56·1 - 81·3	35	**53·2**	36·7 - 69·7	90	**67·8**	57·7 - 78·0	78	**66·5**	55·9 - 77·0
2005-2009	4			67	**77·5**	68·1 - 87·0	40	**70·6**	58·2 - 83·0	21	**52·1**	33·9 - 70·3	66	**63·2**	52·4 - 74·0	66	**70·8**	59·4 - 82·1
Chilean registries																		
1998-1999				9			8						14	*35·8*	*12·6 - 58·9*	3		
2000-2004	1			10	**60·1**	31·6 - 88·5	7			3			13	*77·0*	*55·1 - 99·0*	8		
2005-2008				11	**59·4**	30·5 - 88·3	8			4			9			14	*79·1*	*58·8 - 99·4*
Colombia (Cali)																		
1995-1999	8			55	**34·9**	22·1 - 47·6	42	**59·3**	42·9 - 75·6	19	**26·6**	7·8 - 45·4	73	**37·4**	25·0 - 49·7	51	**49·8** [R]	38·7 - 60·8
2000-2004	5			54	**57·8**	44·4 - 71·1	47	**49·2**	33·9 - 64·4	30	**39·9**	21·4 - 58·3	74	**51·7**	39·5 - 63·9	62	**47·3**	34·8 - 59·8
2005-2009	4			45	**71·9**	58·6 - 85·3	38	**53·6**	34·7 - 72·6	30	**32·1**	15·1 - 49·2	51	**54·2**	40·6 - 67·8	66	**53·3**	41·0 - 65·6

Web-table 2: Precursor-cell lymphoid leukaemia (ICCC3 Ia1) - Five-year net survival (NS, %) by age group and sex: continent, country and calendar period of diagnosis

Calendar	Age group (years)												Sex					
	<1			1-4			5-9			10-14			Boys			Girls		
	N	NS %	95% CI	N	NS %	95% CI	N	NS %	95% CI	N	NS %	95% CI	N	NS %	95% CI	N	NS %	95% CI
Ecuador (Quito)																		
1995-1999	2			25	**64·1**	45·8 - 82·5	33	**66·8**	51·0 - 82·6	21	**57·4**	37·1 - 77·8	41	**58·3** [R]	46·6 - 70·0	40	**70·0**	56·8 - 83·1
2000-2004	5			45	**66·8**	53·2 - 80·5	39	**66·8**	52·2 - 81·4	21	**57·4**	36·8 - 77·9	49	**60·1**	46·4 - 73·9	61	**67·8**	55·6 - 80·0
2005-2009				45	**66·6**	53·8 - 79·4	24	**74·7**	58·6 - 90·7	26	**47·8**	30·2 - 65·5	52	**66·7**	54·5 - 78·9	43	**61·1** [R]	50·6 - 71·5
Puerto Rico *																		
2000-2004	1			32	**81·4**	68·1 - 94·7	14	**85·8**	68·1 - 100·0	26	**69·3**	51·9 - 86·6	40	**68·2** [R]	56·9 - 79·5	33	**90·5** [R]	81·8 - 99·2
2005-2009	5			41	**80·1**	66·8 - 93·4	16	**81·3**	62·8 - 99·8	19	**72·8**	54·7 - 90·9	56	**74·9**	61·6 - 88·2	25	*81·2*	*66·6 - 95·8*
**AMERICA (North)**																		
Canada *																		
1995-1999	35	**54·5**	38·3 - 70·8	604	**91·8**	89·6 - 94·0	326	**88·4**	84·9 - 91·9	169	**79·4**	73·3 - 85·5	634	**84·8**	81·5 - 88·0	500	**87·8**	84·1 - 91·4
2000-2004	31	**51·8**	34·5 - 69·0	545	**95·3**	93·5 - 97·1	320	**91·6**	88·6 - 94·6	178	**88·3**	83·6 - 93·0	626	**89·8**	87·0 - 92·5	448	**92·6**	89·7 - 95·5
2005-2009	31	**48·3**	31·5 - 65·2	571	**94·6**	92·7 - 96·5	320	**93·8**	91·2 - 96·5	175	**86·3**	80·9 - 91·6	628	**90·2**	87·3 - 93·0	469	**91·5**	88·4 - 94·7
US registries																		
1995-1999	259	**55·8**	49·7 - 61·9	3,907	**90·8**	89·9 - 91·8	2,252	**86·6**	85·2 - 88·1	1,252	**73·3**	70·8 - 75·7	4,261	**81·5**	80·1 - 82·9	3,409	**84·6**	83·1 - 86·0
2000-2004	335	**60·0**	54·7 - 65·3	4,519	**93·0**	92·3 - 93·8	2,448	**89·5**	88·3 - 90·8	1,540	**79·6**	77·5 - 81·6	4,855	**86·0**	84·9 - 87·2	3,987	**87·4**	86·2 - 88·6
2005-2009	337	**59·4**	54·1 - 64·7	4,983	**94·4**	93·7 - 95·0	2,688	**90·6**	89·5 - 91·7	1,727	**80·5**	78·5 - 82·4	5,400	**86·9**	85·8 - 88·0	4,335	**88·7**	87·6 - 89·9
**ASIA**																		
Chinese registries																		
1995-1999	1			11	**9·1**	0·0 - 22·5	11	**18·2**	0·0 - 38·0	5			14	*0·0*		14	*17·9*	*0·0 - 36·6*
2000-2004	2			34	**49·0**	32·2 - 65·8	25	**71·1**	53·4 - 88·8	23	**29·0**	10·9 - 47·1	45	**54·4**	40·7 - 68·2	39	**42·4**	30·2 - 54·6
2005-2009	8			58	**84·2**	74·0 - 94·4	45	**76·9**	63·6 - 90·2	40	**52·6**	34·3 - 71·0	92	**73·0**	63·2 - 82·8	59	**63·3**	49·2 - 77·4
Cyprus *																		
2004-2009	1			14	**92·4** [M]	78·4 - 100·0	10	**100·0** [M]	100·0 - 100·0	9			18	*93·4* [M]	*81·2 - 100·0*	16	*73·2* [M]	*51·2 - 95·2*
India (Karunagappally)																		
1995-1999	1			6			8			2			11	*63·9*	*37·0 - 90·9*	6		
2000-2004				6			3			5			9			5		
2005-2009				6			5			1			10	*74·8*	*50·8 - 98·9*	2		
Indonesia (Jakarta)																		
2005-2007				5			5			4			8			6		

Web-table 2: Precursor-cell lymphoid leukaemia (ICCC3 Ia1) - Five-year net survival (NS, %) by age group and sex: continent, country and calendar period of diagnosis

	Age group (years)												Sex					
Calendar	<1			1-4			5-9			10-14			Boys			Girls		
	N	NS %	95% CI	N	NS %	95% CI	N	NS %	95% CI	N	NS %	95% CI	N	NS %	95% CI	N	NS %	95% CI
Israel *																		
1995-1999	7			88	**93·3**	88·0 - 98·5	63	**82·6**	73·3 - 91·9	30	**66·7**	50·2 - 83·2	102	**78·6**	69·7 - 87·5	86	**83·8**	75·0 - 92·6
2000-2004	9			121	**91·8**	86·9 - 96·7	87	**87·4**	80·5 - 94·4	41	**83·0**	71·6 - 94·4	144	**87·6**	81·5 - 93·6	114	**84·1**	76·4 - 91·9
2005-2009	10	**42·4**	16·8 - 68·0	136	**93·2**	88·8 - 97·5	67	**86·8**	79·2 - 94·4	58	**78·2**	67·2 - 89·2	155	**82·5**	75·8 - 89·1	116	**87·5**	80·9 - 94·0
Japanese registries																		
1995-1999	11	**45·6**	18·1 - 73·1	140	**82·8**	76·5 - 89·0	82	**87·9**	80·8 - 94·9	61	**66·2**	54·3 - 78·2	167	**75·7**	68·9 - 82·6	127	**81·2**	73·8 - 88·6
2000-2004	15	**64·4**	40·4 - 88·4	202	**89·8**	85·4 - 94·2	118	**79·1**	71·5 - 86·7	71	**66·7**	55·1 - 78·3	229	**71·6**	64·6 - 78·6	177	**85·8**	79·6 - 91·9
2005-2009	12	**73·8**	49·5 - 98·1	109	**92·4**	88·1 - 96·7	84	**83·8**	76·9 - 90·6	54	**71·6**	60·5 - 82·6	140	**77·3**	70·7 - 84·0	119	**87·8**	82·0 - 93·7
Korea * [‡]																		
1995-1999	49	**34·9**	21·7 - 48·0	528	**74·4**	70·6 - 78·1	353	**67·5**	62·6 - 72·4	241	**51·1**	44·8 - 57·4	674	**60·7**	56·7 - 64·6	497	**66·4**	62·1 - 70·7
2000-2004	48	**41·8**	28·1 - 55·5	514	**84·1**	81·0 - 87·3	383	**76·6**	72·3 - 80·8	252	**62·4**	56·4 - 68·3	724	**72·8**	69·3 - 76·3	473	**73·8**	69·6 - 78·0
2005-2009	53	**46·6**	33·0 - 60·2	460	**84·6**	81·4 - 87·9	326	**79·7**	75·4 - 84·0	298	**70·6**	65·2 - 75·9	630	**77·4**	74·0 - 80·7	507	**76·8**	72·9 - 80·7
Malaysia (Penang)																		
1995-1999	3			22	**82·0**	66·2 - 97·7	16	**87·6**	71·9 - 100·0	10	**60·1**	31·6 - 88·6	32	*78·3*	*64·2 - 92·4*	19	*84·2*	*68·3 - 100·0*
2000-2004	6			25	**92·1**	81·7 - 100·0	16	**87·5**	71·9 - 100·0	4			26	*92·5*	*82·4 - 100·0*	25	*80·0*	*64·7 - 95·4*
2005-2009				25	**84·2**	68·1 - 100·0	17	**81·2**	62·5 - 99·9	15	**42·6**	16·0 - 69·3	36	**82·5** [R]	71·4 - 93·6	21	*61·1*	*40·6 - 81·7*
Mongolia *																		
2005-2009	3			4			11	**0·0**	0·0 - 0·0	6			17	*0·0*	*0·0 - 0·0*	7		
Taiwan *																		
1995-1999	23	**30·6**	12·5 - 48·6	282	**74·6**	69·5 - 79·6	172	**66·9**	59·9 - 73·9	124	**49·3**	40·5 - 58·0	346	**63·5**	58·3 - 68·7	255	**60·9**	54·2 - 67·5
2000-2004	36	**33·5**	18·4 - 48·5	317	**82·7**	78·6 - 86·9	196	**77·1**	71·2 - 83·0	116	**61·3**	52·5 - 70·1	381	**69·1**	64·0 - 74·2	284	**76·1**	70·4 - 81·7
2005-2009	26	**31·5**	15·2 - 47·8	277	**86·5**	82·7 - 90·4	168	**81·5**	75·7 - 87·3	139	**71·2**	63·3 - 79·1	355	**77·2**	72·6 - 81·9	255	**79·4**	74·0 - 84·8
Thai registries																		
1995-1999	8			55	**48·3**	34·4 - 62·1	25	**50·0**	30·2 - 69·7	14	**54·7**	29·0 - 80·3	52	**44·9** [R]	33·1 - 56·6	50	**57·4** [R]	45·1 - 69·7
2000-2004	7			53	**69·5**	57·0 - 82·0	42	**63·8**	49·3 - 78·4	18	**44·6**	22·6 - 66·6	74	**57·1**	45·5 - 68·6	46	**66·5** [R]	55·2 - 77·7
2005-2009	4			36	**62·5**	47·7 - 77·3	32	**47·1**	30·9 - 63·4	30	**53·8**	34·2 - 73·3	58	**52·3**	40·1 - 64·6	44	**58·4**	42·8 - 74·1

Web-table 2: Precursor-cell lymphoid leukaemia (ICCC3 Ia1) - Five-year net survival (NS, %) by age group and sex: continent, country and calendar period of diagnosis

Calendar	Age group (years)												Sex					
	<1			1-4			5-9			10-14			Boys			Girls		
	N	NS %	95% CI	N	NS %	95% CI	N	NS %	95% CI	N	NS %	95% CI	N	NS %	95% CI	N	NS %	95% CI
Turkey (Izmir)																		
1995-1999	5			56	**65·3**	52·4 - 78·3	31	**69·5**	52·2 - 86·8	24	**57·8**	37·3 - 78·2	60	**57·4** [R]	46·6 - 68·2	56	**71·6**	58·8 - 84·5
2000-2004	3			62	**73·9**	62·9 - 84·9	41	**82·5**	70·8 - 94·2	25	**56·6**	36·9 - 76·3	68	**64·2**	51·8 - 76·6	63	**76·2**	64·8 - 87·7
2005-2009	6			78	**80·6**	71·6 - 89·6	49	**84·2**	73·5 - 95·0	28	**62·0**	44·7 - 79·3	83	**74·2**	63·1 - 85·2	78	**72·4**	62·0 - 82·7
**EUROPE**																		
Austria *																		
1995-1999	5			126	**95·3**	91·6 - 99·0	74	**86·5**	78·8 - 94·3	35	**80·1**	67·0 - 93·1	142	**88·3**	81·8 - 94·8	98	**84·1**	75·6 - 92·6
2000-2004	6			98	**97·0**	93·6 - 100·0	57	**87·8**	79·3 - 96·2	47	**85·2**	75·1 - 95·3	110	**92·9**	87·7 - 98·0	98	**86·4**	79·2 - 93·5
2005-2009	9			100	**99·1**	97·1 - 100·0	72	**89·8**	82·7 - 97·0	40	**86·3**	76·1 - 96·4	123	**94·6**	90·1 - 99·1	98	**87·1**	79·2 - 95·0
Belarus *																		
1995-1999	8			112	**83·2**	76·2 - 90·1	96	**80·1**	72·1 - 88·1	66	**63·8**	52·3 - 75·3	156	**70·2**	63·0 - 77·3	126	**81·2**	74·0 - 88·4
2000-2004	2			110	**84·6**	77·9 - 91·4	62	**82·1**	72·6 - 91·7	61	**69·0**	57·5 - 80·5	133	**78·3**	71·0 - 85·6	102	**78·6**	70·5 - 86·7
2005-2009	8			101	**91·1**	85·4 - 96·7	65	**92·0**	85·3 - 98·8	35	**84·2**	73·5 - 95·0	101	**91·2**	85·5 - 96·9	108	**86·1**	79·1 - 93·1
Belgium *																		
2004-2009	12	**33·1** [M]	6·5 - 59·7	191	**93·2** [M]	88·6 - 97·9	105	**86·1** [M]	78·4 - 93·9	78	**84·8** [M]	75·5 - 94·1	230	**87·1** [M]	81·5 - 92·6	156	**87·7** [M]	81·4 - 94·0
Bulgaria *																		
1995-1999	10	**20·2**	0·0 - 41·4	68	**63·4**	52·0 - 74·8	47	**66·0**	52·7 - 79·4	41	**48·9**	33·8 - 63·9	86	**52·0**	42·0 - 62·0	80	**63·2**	52·8 - 73·6
2000-2004	4			67	**76·3**	66·1 - 86·4	53	**58·6**	45·5 - 71·7	30	**53·5**	36·0 - 70·9	86	**61·1**	50·5 - 71·8	68	**62·2**	50·6 - 73·8
2005-2009	6			95	**82·3**	74·1 - 90·6	76	**74·1**	63·3 - 85·0	38	**61·8**	46·5 - 77·0	119	**67·4**	57·9 - 76·9	96	**76·3**	66·4 - 86·3
Croatia *																		
1998-1999	2			20	**90·1**	77·3 - 100·0	14	**78·6**	58·0 - 99·3	8			17	*64·8*	*42·9 - 86·8*	27	*77·9*	*62·6 - 93·2*
2000-2004	5			87	**91·0**	84·9 - 97·0	47	**85·2**	75·1 - 95·2	21	**71·4**	52·7 - 90·2	95	**76·9**	67·0 - 86·8	65	**90·2** [R]	84·2 - 96·3
2005-2009	2			61	**93·8**	87·7 - 99·8	41	**82·5**	70·8 - 94·2	42	**82·3**	70·4 - 94·1	89	**85·0**	77·2 - 92·8	57	**88·0**	79·9 - 96·2
Denmark *																		
1995-1999	1			96	**87·5**	81·0 - 94·1	38	**86·9**	76·3 - 97·5	28	**82·2**	68·3 - 96·1	89	**84·8**	76·7 - 92·9	74	**87·3**	78·5 - 96·0
2000-2004	5			114	**92·2**	87·2 - 97·1	49	**83·7**	73·5 - 94·0	41	**78·1**	65·7 - 90·6	125	**83·2**	75·6 - 90·7	84	**86·3**	78·2 - 94·5
2005-2009	4			92	**93·0**	88·0 - 98·0	55	**84·8**	75·2 - 94·5	20	**85·4**	72·4 - 98·4	97	**85·7**	77·9 - 93·6	74	**89·2**	81·5 - 96·9

Web-table 2: Precursor-cell lymphoid leukaemia (ICCC3 Ia1) - Five-year net survival (NS, %) by age group and sex: continent, country and calendar period of diagnosis

Calendar	Age group (years)												Sex					
	<1			1-4			5-9			10-14			Boys			Girls		
	N	NS %	95% CI	N	NS %	95% CI	N	NS %	95% CI	N	NS %	95% CI	N	NS %	95% CI	N	NS %	95% CI
Estonia *																		
1995-1999	1			11	**72·8**	47·8 - 97·7	10	**30·0**	4·5 - 55·6	7			9			20	*50·1*	*28·9 - 71·2*
2000-2004				13	**75·6**	52·6 - 98·7	7			11	**45·6**	18·1 - 73·0	17	*56·6*	*33·3 - 79·9*	14	*64·4*	*40·4 - 88·4*
2005-2008	1			5			1			6			6			7		
Finland *																		
1995-1999	6			102	**89·3**	83·3 - 95·3	51	**76·5**	65·0 - 88·0	34	**82·4**	69·8 - 95·0	100	**86·9**	79·7 - 94·2	93	**78·0**	68·4 - 87·7
2000-2004	6			117	**90·6**	85·4 - 95·9	44	**88·7**	79·4 - 98·0	24	**75·1**	58·2 - 92·0	102	**81·1**	72·1 - 90·1	89	**90·9** [R]	85·4 - 96·5
2005-2009	14	**69·3**	41·5 - 97·2	99	**87·3**	80·9 - 93·8	59	**81·5**	71·1 - 91·9	33	**78·0**	62·4 - 93·7	118	**79·2**	70·0 - 88·5	87	**84·0**	74·7 - 93·4
France *																		
1995-1999	49	**47·1**	33·3 - 60·9	833	**88·8**	86·6 - 90·9	522	**86·8**	83·9 - 89·7	324	**74·0**	69·2 - 78·8	960	**81·9**	79·2 - 84·6	768	**83·2**	80·2 - 86·2
2000-2004	51	**60·9**	47·6 - 74·2	873	**93·3**	91·6 - 95·0	530	**89·1**	86·4 - 91·7	339	**83·8**	79·9 - 87·8	957	**86·4**	84·0 - 88·8	836	**90·4**	88·1 - 92·7
2005-2009	53	**43·9**	30·0 - 57·9	888	**94·7**	93·2 - 96·2	535	**91·4**	89·0 - 93·8	352	**84·1**	80·1 - 88·0	1,015	**87·2**	84·9 - 89·6	813	**91·9**	89·7 - 94·0
German registries																		
1995-1999	11	**70·2**	43·4 - 97·1	204	**91·7**	87·9 - 95·5	153	**87·6**	82·4 - 92·8	100	**81·1**	73·4 - 88·8	274	**85·0**	80·5 - 89·6	194	**88·5**	83·6 - 93·4
2000-2004	17	**63·5**	40·9 - 86·1	281	**89·4**	85·8 - 93·0	186	**89·8**	85·4 - 94·2	158	**83·6**	77·8 - 89·4	363	**87·0**	83·4 - 90·5	279	**86·9**	82·6 - 91·1
2005-2009	31	**55·4**	36·8 - 74·0	501	**94·0**	91·6 - 96·4	280	**93·1**	89·8 - 96·4	177	**89·7**	85·1 - 94·2	535	**90·3**	87·4 - 93·3	454	**93·2**	90·4 - 96·0
Iceland *																		
1995-1999	1			6			2						6			3		
2000-2004	3			4			1			1			5			4		
2005-2009				6			2			2			4			6		
Ireland *																		
1995-1999	4			78	**84·7**	76·7 - 92·6	39	**87·2**	76·9 - 97·6	25	**68·1**	50·2 - 86·0	78	**82·7**	73·7 - 91·7	68	**76·0**	65·1 - 86·9
2000-2004	4			63	**88·9**	81·2 - 96·6	43	**86·1**	75·9 - 96·3	35	**77·2**	63·5 - 90·9	79	**86·1**	78·3 - 94·0	66	**79·2**	69·3 - 89·0
2005-2009	10	**56·1**	25·4 - 86·8	91	**97·1**	93·1 - 100·0	34	**87·6**	77·6 - 97·7	27	**73·9**	58·6 - 89·2	93	**88·9**	81·6 - 96·2	69	**79·1**	68·5 - 89·8
Italian registries																		
1995-1999	21	**43·0**	22·6 - 63·4	302	**89·8**	86·4 - 93·2	214	**85·8**	81·1 - 90·6	124	**78·5**	71·2 - 85·8	358	**83·1**	78·7 - 87·4	303	**84·6**	80·1 - 89·0
2000-2004	33	**69·4**	53·8 - 85·0	375	**94·4**	92·1 - 96·8	203	**87·7**	83·2 - 92·2	114	**67·3**	58·7 - 75·9	411	**80·8**	76·5 - 85·2	314	**85·2**	80·1 - 90·2
2005-2009	13	**51·9**	30·3 - 73·6	257	**94·2**	91·7 - 96·7	148	**85·1**	80·0 - 90·2	89	**87·0**	80·3 - 93·6	269	**84·4**	79·8 - 89·0	238	**92·1**	88·7 - 95·4

Web-table 2: Precursor-cell lymphoid leukaemia (ICCC3 Ia1) - Five-year net survival (NS, %) by age group and sex: continent, country and calendar period of diagnosis

Calendar	Age group (years)												Sex					
	<1			1-4			5-9			10-14			Boys			Girls		
	N	NS %	95% CI	N	NS %	95% CI	N	NS %	95% CI	N	NS %	95% CI	N	NS %	95% CI	N	NS %	95% CI
Latvia *																		
1995-1999	2			5			3			5			6			9		
2000-2004				21	**95·4**	86·5 - 100·0	8			6			18	*89·1*	*75·0 - 100·0*	17	*94·1*	*83·3 - 100·0*
2005-2009				24	**87·1**	73·6 - 100·0	14	**71·9**	45·9 - 97·8	7			24	**71·5** [R]	56·6 - 86·5	21	*89·3*	*75·5 - 100·0*
Lithuania *																		
1995-1999	1			54	**57·5**	44·5 - 70·5	29	**69·0**	52·5 - 85·5	18	**50·2**	27·9 - 72·4	65	**61·1**	49·1 - 73·0	37	**58·2** [R]	45·2 - 71·3
2000-2004	3			44	**81·9**	70·6 - 93·2	41	**75·3**	62·1 - 88·5	18	**66·7**	45·7 - 87·8	53	**81·8** [R]	73·5 - 90·0	53	**69·1** [R]	59·4 - 78·9
2005-2009	2			46	**91·1**	82·8 - 99·4	27	**74·4**	58·3 - 90·5	11	**44·2**	18·6 - 69·7	36	*79·4*	*65·9 - 92·9*	50	**70·6** [R]	60·7 - 80·6
Malta *																		
1995-1999				9			7			3			12	*66·7*	*41·4 - 92·1*	7		
2000-2004				5			8			3			11	*81·9*	*60·2 - 100·0*	5		
2005-2009				10	**90·2**	72·5 - 100·0	3			4			8			9		
Netherlands *																		
1995-1999	17	**35·4**	13·8 - 56·9	281	**88·0**	84·1 - 91·8	143	**82·3**	76·0 - 88·6	86	**75·7**	66·6 - 84·7	335	**81·2**	76·4 - 86·0	192	**80·2**	73·5 - 86·9
2000-2004	24	**62·7**	43·7 - 81·6	290	**92·4**	89·4 - 95·5	159	**89·4**	84·6 - 94·1	109	**72·5**	64·2 - 80·9	347	**83·2**	78·9 - 87·5	235	**85·1**	79·7 - 90·5
2005-2009	23	**67·0**	47·4 - 86·6	245	**89·4**	85·7 - 93·1	180	**89·9**	85·3 - 94·4	85	**81·1**	73·0 - 89·1	291	**86·1**	81·7 - 90·4	242	**86·6**	81·4 - 91·8
Norway *																		
1995-1999	6			96	**88·4**	82·0 - 94·9	54	**79·7**	69·0 - 90·3	21	**70·5**	51·1 - 89·8	88	**77·3**	66·9 - 87·6	89	**81·0**	70·1 - 91·9
2000-2004	4			97	**86·3**	79·4 - 93·2	47	**89·4**	80·7 - 98·1	30	**86·7**	74·8 - 98·7	94	**86·5**	78·6 - 94·3	84	**89·1**	82·3 - 96·0
2005-2009	9			94	**92·0**	86·3 - 97·7	41	**93·5**	86·4 - 100·0	25	**85·9**	73·3 - 98·6	84	**91·4**	85·1 - 97·7	85	**88·2**	80·2 - 96·2
Poland (Wroclaw)																		
2000-2004	2			15	**80·1**	60·5 - 99·6	6			10	**50·0**	21·2 - 78·8	18	*61·2*	*39·4 - 82·9*	15	*80·0*	*60·5 - 99·5*
2005-2009	2			25	**89·8**	76·4 - 100·0	18	**86·2**	69·0 - 100·0	17	**65·9**	44·5 - 87·3	44	**78·0**	65·2 - 90·8	18	*88·3*	*73·3 - 100·0*
Portugal *																		
1998-1999	4			22	**76·3**	58·5 - 94·0	12	**63·7**	36·8 - 90·5	7			20	*68·5*	*48·2 - 88·8*	25	*62·7*	*43·8 - 81·5*
2000-2004	10	**90·5**	72·8 - 100·0	110	**89·0**	83·1 - 94·9	71	**84·5**	76·1 - 92·9	47	**63·9**	50·4 - 77·5	131	**77·8**	70·1 - 85·4	107	**80·7**	72·5 - 89·0
2005-2009	5			87	**91·0**	85·4 - 96·7	66	**94·8**	89·7 - 99·8	33	**68·4**	53·8 - 82·9	117	**81·4**	73·9 - 88·9	74	**86·0**	77·8 - 94·1

Web-table 2: Precursor-cell lymphoid leukaemia (ICCC3 Ia1) - Five-year net survival (NS, %) by age group and sex: continent, country and calendar period of diagnosis

Calendar	Age group (years)												Sex					
	<1			1-4			5-9			10-14			Boys			Girls		
	N	NS %	95% CI	N	NS %	95% CI	N	NS %	95% CI	N	NS %	95% CI	N	NS %	95% CI	N	NS %	95% CI
Russia ‡ (Arkhangelsk)																		
2000-2004	1			12	**66·7**	41·4 - 92·1	3			7			14	*42·9*	*18·5 - 67·4*	9		
2005-2009				11	**80·1**	56·6 - 100·0	7			5			14	*77·1*	*55·1 - 99·1*	9		
Slovakia *																		
2000-2004	4			59	**88·2**	80·0 - 96·4	39	**84·7**	73·5 - 95·8	29	**69·1**	52·6 - 85·6	69	**85·5**	77·0 - 94·1	62	**71·8**	60·1 - 83·6
2005-2007	3			44	**92·2**	83·8 - 100·0	24	**83·9**	69·6 - 98·2	18	**60·6**	40·1 - 81·2	56	**78·3**	66·9 - 89·8	33	*82·4*	*69·8 - 95·1*
Slovenia *																		
1995-1999	3			25	**92·1**	81·6 - 100·0	9			13	**77·0**	55·0 - 98·9	25	*76·1*	*59·7 - 92·5*	25	*92·0*	*81·6 - 100·0*
2000-2004	1			32	**96·9**	91·0 - 100·0	12	**91·7**	76·7 - 100·0	4			22	*95·5*	*87·0 - 100·0*	27	*88·9*	*77·3 - 100·0*
2005-2009	3			24	**91·7**	80·9 - 100·0	8			8			22	*81·9*	*66·1 - 97·6*	21	*81·8*	*66·0 - 97·6*
Spanish registries																		
1995-1999	11	**27·4**	3·5 - 51·2	142	**85·1**	79·2 - 91·0	86	**82·6**	74·6 - 90·6	51	**58·9**	45·5 - 72·2	168	**74·7**	67·5 - 82·0	122	**73·4**	65·0 - 81·7
2000-2004	12	**58·5**	32·0 - 84·9	149	**83·2**	77·2 - 89·2	79	**84·9**	77·0 - 92·7	68	**79·4**	69·8 - 89·0	174	**84·4**	78·8 - 90·0	134	**78·3**	70·6 - 85·9
2005-2009	14	**85·4**	67·0 - 100·0	163	**90·8**	86·4 - 95·3	125	**88·4**	82·7 - 94·1	60	**73·7**	63·3 - 84·0	219	**85·3**	80·1 - 90·5	143	**82·6**	75·6 - 89·6
Sweden *																		
1995-1999	12	**50·1**	23·4 - 76·7	184	**92·9**	89·2 - 96·6	95	**85·3**	78·2 - 92·4	53	**79·3**	68·5 - 90·1	197	**83·9**	78·1 - 89·7	147	**87·1**	80·3 - 93·9
2000-2004	10	**50·1**	21·3 - 78·9	143	**95·1**	91·6 - 98·7	98	**85·7**	78·8 - 92·6	62	**82·3**	72·9 - 91·8	175	**88·1**	83·1 - 93·1	138	**84·4**	77·3 - 91·6
2005-2009	7			118	**90·0**	84·8 - 95·2	61	**85·3**	77·3 - 93·3	44	**83·6**	73·9 - 93·4	122	**86·0**	80·1 - 91·9	108	**85·1**	77·9 - 92·4
Switzerland *																		
1995-1999	2			116	**93·2**	88·5 - 97·8	59	**89·8**	82·0 - 97·5	43	**74·5**	61·6 - 87·4	135	**81·7**	74·7 - 88·8	85	**92·8**	86·7 - 98·8
2000-2004	8			104	**91·4**	86·0 - 96·8	72	**88·9**	81·7 - 96·1	38	**84·3**	72·8 - 95·7	122	**85·8**	78·8 - 92·8	100	**88·9**	82·3 - 95·4
2005-2009	9			113	**89·8**	84·1 - 95·5	52	**91·0**	83·5 - 98·5	51	**86·7**	76·8 - 96·6	127	**89·5**	83·7 - 95·3	98	**86·3**	78·8 - 93·9
United Kingdom *																		
1995-1999	62	**37·8**	25·8 - 49·9	968	**87·9**	85·9 - 90·0	531	**84·5**	81·4 - 87·6	310	**68·0**	62·8 - 73·2	1,038	**78·3**	75·5 - 81·2	833	**80·3**	77·0 - 83·5
2000-2004	76	**63·1**	52·3 - 74·0	953	**92·1**	90·3 - 93·8	569	**89·0**	86·4 - 91·5	356	**79·2**	75·0 - 83·5	1,115	**85·1**	82·7 - 87·5	839	**87·4**	84·8 - 89·9
2005-2009	63	**57·8**	46·5 - 69·1	1,001	**93·5**	91·9 - 95·0	490	**91·5**	89·2 - 93·9	339	**85·1**	81·3 - 88·9	1,036	**87·7**	85·4 - 90·0	857	**91·2**	89·0 - 93·4

Web-table 2: Precursor-cell lymphoid leukaemia (ICCC3 Ia1) - Five-year net survival (NS, %) by age group and sex: continent, country and calendar period of diagnosis

Calendar	Age group (years)												Sex					
	<1			1-4			5-9			10-14			Boys			Girls		
	N	NS %	95% CI	N	NS %	95% CI	N	NS %	95% CI	N	NS %	95% CI	N	NS %	95% CI	N	NS %	95% CI
**OCEANIA**																		
Australian registries																		
1995-1999	17	**41·3**	19·0 - 63·6	340	**88·6**	85·2 - 92·0	204	**85·3**	80·5 - 90·2	121	**76·9**	69·5 - 84·4	363	**80·3**	75·7 - 84·9	319	**85·9**	81·6 - 90·2
2000-2004	28	**60·7**	42·9 - 78·5	425	**92·0**	89·4 - 94·7	235	**84·6**	80·0 - 89·2	137	**83·8**	77·6 - 90·0	445	**82·5**	78·4 - 86·6	380	**90·5**	87·1 - 94·0
2005-2009	12	**62·7**	39·9 - 85·5	327	**95·0**	92·7 - 97·3	156	**92·1**	88·3 - 96·0	122	**81·3**	74·3 - 88·4	352	**90·3**	86·8 - 93·7	265	**86·6**	81·8 - 91·3
New Zealand *																		
1995-1999	4			89	**90·0**	83·7 - 96·2	45	**80·1**	68·5 - 91·6	29	**79·4**	64·9 - 93·9	104	**85·1**	77·4 - 92·8	63	**78·4**	67·1 - 89·6
2000-2004	5			93	**94·7**	90·1 - 99·2	52	**90·4**	82·5 - 98·4	32	**75·1**	60·4 - 89·9	103	**92·0**	85·7 - 98·3	79	**78·8**	69·2 - 88·4
2005-2009	6			77	**94·8**	89·8 - 99·8	62	**87·1**	78·1 - 96·0	31	**87·9**	75·3 - 100·0	93	**92·5**	86·3 - 98·7	83	**86·1**	77·5 - 94·7

Web-table 3

Acute myeloid leukaemia (ICCC-3 Ib) - Five-year net survival (NS, %) by age group and sex: continent, country and calendar period of diagnosis

Net survival estimates by sex were age-standardised where possible. Estimates in italics are not age-standardised

* National coverage – the data are derived from a population-based cancer registry (registries) covering the entire country

[M] Estimate based on merging data for 2 (or all 3) calendar periods

[R] Age-standardised estimate computed by pooling two age-specific estimates and re-estimation

[§] Estimate judged as less reliable

‡ Korea: Republic of Korea; Russia: Russian Federation

Web-table 3: Acute myeloid leukaemia (ICCC3 Ib) - Five-year net survival (NS, %) by age group and sex: continent, country and calendar period of diagnosis

	Age group (years)												Sex					
Calendar	<1			1-4			5-9			10-14			Boys			Girls		
	N	NS %	95% CI	N	NS %	95% CI	N	NS %	95% CI	N	NS %	95% CI	N	NS %	95% CI	N	NS %	95% CI
**AFRICA**																		
Algerian registries																		
2000-2004	2			3			5			3			11	*16·0* [§]	*0·0 - 38·0*	2		
2005-2009	1			4			1			1			3			4		
Libya (Benghazi)																		
2003-2004				5									1			4		
Tunisia (Central)																		
1996-1999				2			1						2			1		
2000-2004	1						1						2					
2005-2007				2						1			2			1		
**AMERICA (Central and South)**																		
Brazilian registries																		
1996-1999	1			5			4			3			7			6		
2000-2004	5			11	**27·4**	3·7 - 51·1	15	**66·7**	43·8 - 89·6	10	**40·1**	12·2 - 68·0	28	**45·4** [R]	33·0 - 57·8	13	*54·1*	*28·3 - 79·9*
2005-2009	3			5			8			9			17	*47·0*	*27·3 - 66·7*	8		
Chilean registries																		
1998-1999				2						1			2			1		
2000-2004										2			2					
2005-2008	1			4			3			1			8			1		
Ecuador (Quito)																		
1995-1999	3			5			12	**41·7**	15·7 - 67·7	13	**46·3**	20·7 - 71·9	16	*62·9*	*40·0 - 85·8*	17	*35·5*	*14·1 - 57·0*
2000-2004	2			5			4			7			10	*50·3*	*21·4 - 79·1*	8		
2005-2009	3			7			1			5			8			8		
Puerto Rico *																		
2000-2004	1			4			6			6			10	*60·1*	*31·6 - 88·5*	7		
2005-2009	2			5			4			7			8			10	*44·4*	*16·9 - 71·8*

Web-table 3: Acute myeloid leukaemia (ICCC3 Ib) - Five-year net survival (NS, %) by age group and sex: continent, country and calendar period of diagnosis

Calendar	Age group (years)												Sex					
	<1			1-4			5-9			10-14			Boys			Girls		
	N	NS %	95% CI	N	NS %	95% CI	N	NS %	95% CI	N	NS %	95% CI	N	NS %	95% CI	N	NS %	95% CI
**AMERICA (North)**																		
Canada *																		
1995-1999	31	**61·6**	44·8 - 78·5	80	**56·3**	45·5 - 67·1	51	**56·9**	43·5 - 70·3	73	**53·5**	42·2 - 64·8	118	**53·9**	44·7 - 63·1	117	**58·0**	48·8 - 67·2
2000-2004	28	**57·4**	39·6 - 75·2	58	**69·0**	57·3 - 80·8	48	**56·3**	42·5 - 70·1	54	**70·4**	58·4 - 82·5	85	**65·9**	55·9 - 75·9	103	**63·5**	53·9 - 73·0
2005-2009	26	**69·2**	51·4 - 86·9	67	**78·8**	68·6 - 89·1	42	**68·9**	55·4 - 82·3	65	**70·3**	58·4 - 82·1	110	**70·5**	61·2 - 79·8	90	**74·3**	65·2 - 83·4
US registries																		
1995-1999	193	**45·4**	38·3 - 52·5	547	**54·4**	50·2 - 58·6	336	**55·7**	50·4 - 61·0	498	**46·9**	42·5 - 51·3	854	**52·1**	48·6 - 55·7	720	**50·7**	46·9 - 54·5
2000-2004	262	**57·6**	51·6 - 63·6	602	**62·6**	58·8 - 66·5	369	**60·4**	55·4 - 65·4	566	**57·7**	53·6 - 61·8	976	**59·9**	56·6 - 63·2	823	**59·6**	56·1 - 63·1
2005-2009	295	**60·6**	55·0 - 66·2	650	**66·2**	62·5 - 69·9	354	**63·5**	58·5 - 68·5	599	**61·8**	57·9 - 65·7	976	**63·3**	60·1 - 66·6	922	**63·1**	59·7 - 66·5
**ASIA**																		
Chinese registries																		
1995-1999				6			10	**10·0**	0·0 - 24·5	11	**0·0**		18	*5·6*	*0·0 - 13·9*	9		
2000-2004	2			8			18	**27·5**	6·4 - 48·6	33	**15·2**	3·6 - 26·7	39	**15·9** R	6·9 - 24·8	22	*26·0*	*8·3 - 43·8*
2005-2009	3			13	**33·0**	6·5 - 59·6	20	**46·0**	22·5 - 69·5	25	**39·5**	19·7 - 59·2	36	**38·8**	22·5 - 55·1	25	*44·0*	*24·2 - 63·8*
Cyprus *																		
2004-2009	1			2			2			5			6			4		
India (Karunagappally)																		
1995-1999										1			1					
2000-2004							2			2			1			3		
2005-2009				1			1			1			3					
Indonesia (Jakarta)																		
2005-2007							3			3			3			3		
Israel *																		
1995-1999	2			13	**53·9**	28·2 - 79·6	9			16	**56·3**	33·0 - 79·6	26	**61·7** R	47·4 - 76·1	14	*64·3*	*40·3 - 88·3*
2000-2004	8			20	**70·1**	50·8 - 89·4	14	**78·6**	57·9 - 99·2	25	**56·0**	37·1 - 75·0	30	**70·4** R	57·7 - 83·1	37	**63·5**	49·5 - 77·6
2005-2009	11	**54·7**	27·0 - 82·4	28	**65·3**	47·2 - 83·3	16	**83·6**	67·1 - 100·0	23	**53·2**	33·5 - 72·8	41	**64·0** R	51·6 - 76·3	37	**66·0** R	54·5 - 77·5

Web-table 3: Acute myeloid leukaemia (ICCC3 Ib) - Five-year net survival (NS, %) by age group and sex: continent, country and calendar period of diagnosis

	Age group (years)												Sex					
Calendar	<1			1-4			5-9			10-14			Boys			Girls		
	N	NS %	95% CI	N	NS %	95% CI	N	NS %	95% CI	N	NS %	95% CI	N	NS %	95% CI	N	NS %	95% CI
Japanese registries																		
1995-1999	19	**62·9**	41·7 - 84·2	27	**55·6**	37·3 - 73·9	28	**67·9**	51·0 - 84·8	48	**60·5**	46·8 - 74·1	71	**61·4**	50·4 - 72·4	51	**62·3** [R]	51·9 - 72·7
2000-2004	24	**72·9**	54·7 - 91·1	69	**69·0**	57·8 - 80·2	48	**56·9**	42·6 - 71·3	40	**56·7**	41·4 - 71·9	96	**63·3**	53·1 - 73·6	85	**59·1**	47·8 - 70·5
2005-2009	13	**70·8**	50·0 - 91·7	37	**76·2**	64·8 - 87·5	31	**62·9**	49·1 - 76·6	30	**70·3**	55·8 - 84·8	57	**73·7**	64·2 - 83·3	54	**65·7**	54·8 - 76·7
Korea * ‡																		
1995-1999	44	**22·8**	10·8 - 34·9	151	**45·8**	37·8 - 53·7	120	**44·2**	35·4 - 53·0	167	**35·4**	28·2 - 42·6	283	**39·9**	34·1 - 45·6	199	**40·3**	33·4 - 47·1
2000-2004	37	**51·5**	35·7 - 67·4	146	**48·0**	39·9 - 56·1	149	**51·0**	43·1 - 59·0	172	**49·5**	42·0 - 56·9	283	**46·4**	40·6 - 52·1	221	**53·8**	47·2 - 60·4
2005-2009	49	**47·4**	33·6 - 61·2	95	**55·5**	46·0 - 65·1	117	**53·7**	45·0 - 62·5	172	**55·5**	48·2 - 62·8	243	**53·4**	47·3 - 59·6	190	**54·4**	47·4 - 61·3
Malaysia (Penang)																		
1995-1999	1			10	**60·0**	31·6 - 88·5	4			7			13	*53·9*	*28·2 - 79·6*	9		
2000-2004	2			9			8			4			14	*71·5*	*48·8 - 94·2*	9		
2005-2009	3			7			8			4			8			14	*33·5*	*10·3 - 56·7*
Mongolia *																		
2005-2009				3			2			3			3			5		
Taiwan *																		
1995-1999	31	**32·4**	16·4 - 48·4	53	**41·6**	28·5 - 54·6	46	**45·7**	31·5 - 59·8	65	**38·5**	26·8 - 50·2	102	**40·0**	30·2 - 49·7	93	**42·1**	32·1 - 52·1
2000-2004	21	**38·3**	18·3 - 58·3	71	**49·3**	37·8 - 60·9	49	**51·0**	37·3 - 64·8	73	**48·0**	36·7 - 59·4	122	**47·9**	39·0 - 56·9	92	**49·7**	39·6 - 59·8
2005-2009	23	**52·8**	33·8 - 71·9	56	**60·3**	47·6 - 73·1	59	**60·3**	47·8 - 72·8	67	**48·7**	37·0 - 60·4	116	**56·3**	47·4 - 65·1	89	**54·2**	43·9 - 64·4
Thai registries																		
1995-1999	3			5			5			5			9			9		
2000-2004	1			8			11	**36·5**	10·3 - 62·7	11	**31·2**	5·2 - 57·3	18	*36·0*	*14·3 - 57·7*	13	*23·1*	*2·4 - 43·9*
2005-2009	7			12	**17·9**	0·0 - 37·5	6			11	**57·1**	30·0 - 84·2	18	*35·5*	*15·1 - 55·9*	18	*53·1*	*28·2 - 78·0*
Turkey (Izmir)																		
1995-1999	7			7			13	**61·6**	36·4 - 86·7	7			16	*43·8*	*20·7 - 67·0*	18	*75·2*	*54·7 - 95·8*
2000-2004	2			8			8			6			14	*39·8*	*14·8 - 64·8*	10	*20·1*	*0·0 - 41·6*
2005-2009	4			6			4			14	**35·8**	12·3 - 59·3	15	*47·1*	*21·2 - 73·0*	13	*40·0*	*13·2 - 66·7*

Web-table 3: Acute myeloid leukaemia (ICCC3 Ib) - Five-year net survival (NS, %) by age group and sex: continent, country and calendar period of diagnosis

Calendar	Age group (years)												Sex					
	<1			1-4			5-9			10-14			Boys			Girls		
	N	NS %	95% CI	N	NS %	95% CI	N	NS %	95% CI	N	NS %	95% CI	N	NS %	95% CI	N	NS %	95% CI
**EUROPE**																		
Austria *																		
1995-1999	1			21	**61·9**	41·8 - 82·1	4			21	**57·2**	36·8 - 77·6	29	**58·7** [R]	44·9 - 72·5	18	*61·1*	*39·5 - 82·7*
2000-2004	6			10	**60·0**	31·6 - 88·4	9			15	**66·7**	43·8 - 89·6	21	*66·8*	*47·2 - 86·4*	19	*63·2*	*42·2 - 84·2*
2005-2009	2			12	**77·8**	52·3 - 100·0	9			12	**66·4**	43·4 - 89·4	16	*87·6*	*71·9 - 100·0*	19	*59·7*	*38·8 - 80·5*
Belgium *																		
2004-2009	10	**53·5** [M]	22·0 - 85·0	24	**42·2** [M]	20·6 - 63·9	17	**63·7** [M]	39·2 - 88·3	17	**60·2** [M]	35·9 - 84·5	31	*48·5* [M]	*29·4 - 67·7*	37	**58·5** [M]	44·5 - 72·6
Bulgaria *																		
1995-1999	1			9			8			10	**50·1**	21·3 - 78·9	17	*23·6*	*4·9 - 42·3*	11	*36·4*	*10·2 - 62·6*
2000-2004	4			10	**30·1**	4·5 - 55·7	7			14	**21·5**	2·0 - 41·0	23	*30·5*	*12·5 - 48·5*	12	*8·4*	*0·0 - 20·7*
2005-2009	3			8			10	**33·8**	6·0 - 61·6	12	**46·5**	18·3 - 74·7	16	*34·1*	*14·6 - 53·6*	17	*24·7*	*2·4 - 46·9*
Croatia *																		
1998-1999	1			7			2			3			8			5		
2000-2004	2			7			7			4			13	*61·6*	*36·4 - 86·7*	7		
2005-2009	2			12	**64·4**	37·8 - 91·0	7			7			15	*53·2*	*28·5 - 78·0*	13	*58·4*	*32·0 - 84·7*
Denmark *																		
1995-1999	4			11	**72·8**	48·8 - 96·7	8			9			13	*53·9*	*28·7 - 79·0*	19	*63·2*	*42·2 - 84·2*
2000-2004	7			22	**68·2**	49·3 - 87·1	5			8			19	*63·2*	*42·2 - 84·2*	23	*74·0*	*56·6 - 91·3*
2005-2009	6			18	**83·8**	67·4 - 100·0	9			5			17	*63·2*	*40·7 - 85·7*	21	*73·0*	*55·3 - 90·7*
Estonia *																		
1995-1999				1			2			4			6			1		
2000-2004	2						1			8			6			5		
2005-2008	3			2						1			3			3		
Finland *																		
1995-1999	4			15	**86·7**	70·1 - 100·0	9			9			19	*68·5*	*48·2 - 88·7*	18	*88·9*	*74·8 - 100·0*
2000-2004	4			10	**70·0**	43·3 - 96·8	3			12	**66·7**	41·4 - 92·1	15	*53·4*	*29·3 - 77·5*	14	*78·6*	*58·0 - 99·3*
2005-2009	3			15	**75·5**	55·3 - 95·6	9			8			16	*73·7*	*52·4 - 95·1*	19	*66·5*	*46·9 - 86·1*

Web-table 3: Acute myeloid leukaemia (ICCC3 Ib) - Five-year net survival (NS, %) by age group and sex: continent, country and calendar period of diagnosis

Calendar	Age group (years)												Sex					
	<1			1-4			5-9			10-14			Boys			Girls		
	N	NS %	95% CI	N	NS %	95% CI	N	NS %	95% CI	N	NS %	95% CI	N	NS %	95% CI	N	NS %	95% CI
France *																		
1995-1999	61	**55·9**	43·6 - 68·3	120	**67·6**	59·2 - 75·9	89	**66·0**	56·2 - 75·9	110	**51·0**	41·7 - 60·3	187	**58·2**	51·0 - 65·3	193	**62·5**	55·4 - 69·7
2000-2004	56	**47·7**	34·7 - 60·7	127	**52·0**	43·4 - 60·6	87	**79·3**	70·9 - 87·8	122	**57·2**	48·4 - 66·0	201	**65·4**	59·1 - 71·7	191	**59·1**	52·1 - 66·1
2005-2009	51	**72·8**	60·6 - 85·0	109	**57·1**	48·1 - 66·1	75	**83·2**	74·9 - 91·5	102	**63·1**	53·6 - 72·6	175	**68·6**	61·8 - 75·3	162	**70·5**	63·6 - 77·5
German registries																		
1995-1999	15	**59·5**	35·3 - 83·7	25	**68·1**	50·2 - 85·9	27	**66·7**	49·4 - 84·0	40	**52·5**	37·4 - 67·7	49	**54·7**	42·0 - 67·4	58	**69·9**	58·5 - 81·2
2000-2004	19	**63·4**	42·3 - 84·4	37	**78·4**	65·4 - 91·5	27	**74·1**	57·9 - 90·3	56	**65·6**	53·2 - 78·0	77	**70·6**	60·6 - 80·6	62	**70·4**	58·5 - 82·4
2005-2009	31	**59·6**	41·4 - 77·9	62	**85·3**	75·8 - 94·8	38	**84·0**	72·4 - 95·6	59	**73·7**	62·8 - 84·7	107	**76·0**	67·7 - 84·4	83	**80·8**	71·8 - 89·7
Iceland *																		
2000-2004				1			1									2		
2005-2009				1						3			2			2		
Ireland *																		
1995-1999	2			11	**63·7**	36·8 - 90·5	7			9			14	*71·5*	*48·8 - 94·2*	15	*60·0*	*36·3 - 83·8*
2000-2004	5			14	**78·6**	58·0 - 99·3	9			7			15	*60·0*	*36·3 - 83·8*	20	*60·1*	*39·3 - 80·8*
2005-2009	7			13	**64·4**	40·4 - 88·3	7			8			19	*69·2*	*49·3 - 89·2*	16	*62·7*	*41·6 - 83·8*
Italian registries																		
1995-1999	17	**51·1**	28·4 - 73·8	30	**61·6**	44·1 - 79·1	25	**68·0**	50·2 - 85·8	37	**56·8**	41·1 - 72·5	52	**58·4**	45·2 - 71·5	57	**64·7**	52·6 - 76·7
2000-2004	7			39	**66·4**	51·8 - 81·1	32	**74·3**	59·2 - 89·4	46	**67·5**	54·1 - 80·8	72	**66·7**	56·2 - 77·2	52	**67·2**	54·8 - 79·6
2005-2009	13	**50·6**	23·5 - 77·7	25	**76·0**	60·8 - 91·2	26	**69·2**	52·7 - 85·6	39	**69·9**	56·4 - 83·3	65	**76·6**	66·7 - 86·6	38	**58·3** [R]	47·4 - 69·2
Latvia *																		
1995-1999	1			4			6			4			7			8		
2000-2004				2			3			6			7			4		
2005-2009	2			2			5						6			3		
Lithuania *																		
1995-1999	3			1			3			5			7			5		
2000-2004	1			11	**9·1**	0·0 - 22·4	8			7			15	*26·7*	*6·0 - 47·5*	12	*16·7*	*0·0 - 35·0*
2005-2009	2			4			2			6			7			7		

Web-table 3: Acute myeloid leukaemia (ICCC3 Ib) - Five-year net survival (NS, %) by age group and sex: continent, country and calendar period of diagnosis

Calendar	Age group (years)												Sex					
	<1			1-4			5-9			10-14			Boys			Girls		
	N	NS %	95% CI	N	NS %	95% CI	N	NS %	95% CI	N	NS %	95% CI	N	NS %	95% CI	N	NS %	95% CI
Malta *																		
2000-2004				1			3						3			1		
2005-2009	1						1			2			2			2		
Netherlands *																		
1995-1999	9			39	**71·8**	57·9 - 85·8	18	**55·6**	33·5 - 77·7	26	**57·7**	39·3 - 76·2	48	**57·9**	43·7 - 72·2	44	**59·1** [R]	47·5 - 70·6
2000-2004	12	**50·1**	23·5 - 76·8	43	**62·8**	48·6 - 77·0	17	**47·1**	24·6 - 69·6	37	**54·1**	38·4 - 69·8	66	**61·0** [R]	51·7 - 70·2	43	**49·1** [R]	37·3 - 60·9
2005-2009	18	**51·4**	29·7 - 73·0	38	**57·8**	42·3 - 73·4	22	**57·4**	34·1 - 80·6	28	**62·5**	46·1 - 78·9	63	**59·9**	47·9 - 71·9	43	**62·7** [R]	50·9 - 74·5
Norway *																		
1995-1999	5			17	**76·5**	57·0 - 96·0	10	**50·0**	21·2 - 78·8	10	**40·1**	12·2 - 67·9	24	*66·7*	*48·4 - 85·1*	18	*50·0*	*28·0 - 72·1*
2000-2004	5			16	**62·5**	39·7 - 85·3	10	**70·0**	43·3 - 96·7	6			20	*75·1*	*56·6 - 93·5*	17	*58·8*	*36·4 - 81·3*
2005-2009	7			11	**73·4**	48·8 - 98·0	5			6			18	*74·9*	*53·9 - 95·8*	11	*58·2*	*32·4 - 84·1*
Poland (Wroclaw)																		
2000-2004				3			4			1			2			6		
2005-2009	2			6			4			4			7			9		
Portugal *																		
1998-1999				5			6			6			9			8		
2000-2004	2			18	**50·0**	27·9 - 72·2	24	**62·6**	43·7 - 81·4	21	**52·4**	31·8 - 73·1	33	*60·7*	*44·3 - 77·0*	32	**47·0** [R]	33·7 - 60·3
2005-2009	1			15	**48·1**	25·9 - 70·3	15	**66·8**	45·7 - 87·9	21	**67·3**	48·0 - 86·6	26	*58·1*	*40·5 - 75·6*	26	*63·0*	*46·1 - 79·9*
Slovakia *																		
2000-2004	1			11	**27·3**	3·6 - 51·0	10	**40·0**	12·2 - 67·9	13	**61·6**	36·4 - 86·7	12	*33·4*	*8·8 - 58·0*	23	*52·2*	*32·4 - 72·0*
2005-2007	6			7			5			6			15	*52·9*	*21·5 - 84·3*	9		
Slovenia *																		
1995-1999				1			3			3			4			3		
2000-2004	1			4			1			5			7			4		
2005-2009	2			6			4			3			3			12	*81·6*	*59·4 - 100·0*
Spanish registries																		
1995-1999	8			15	**40·0**	16·6 - 63·5	4			11	**45·5**	18·1 - 72·9	16	*50·1*	*26·7 - 73·5*	22	*45·5*	*25·4 - 65·7*
2000-2004	5			13	**53·9**	28·2 - 79·5	11	**81·8**	60·2 - 100·0	10	**60·1**	31·6 - 88·5	22	*50·1*	*29·8 - 70·3*	17	*76·5*	*57·0 - 96·0*
2005-2009	4			14	**58·8**	36·5 - 81·2	10	**55·7**	28·5 - 82·9	18	**68·3**	47·9 - 88·6	31	**60·4** [R]	48·0 - 72·7	15	*58·1*	*35·5 - 80·7*

Web-table 3: Acute myeloid leukaemia (ICCC3 Ib) - Five-year net survival (NS, %) by age group and sex: continent, country and calendar period of diagnosis

	Age group (years)												Sex					
Calendar	<1			1-4			5-9			10-14			Boys			Girls		
	N	NS %	95% CI	N	NS %	95% CI	N	NS %	95% CI	N	NS %	95% CI	N	NS %	95% CI	N	NS %	95% CI
Sweden *																		
1995-1999	8			23	**73·9**	56·4 - 91·4	8			11	**72·7**	47·8 - 97·7	20	*75·1*	*56·6 - 93·5*	30	*70·0*	*54·0 - 86·1*
2000-2004	14	**71·5**	49·7 - 93·2	20	**50·0**	28·9 - 71·1	9			13	**38·5**	14·0 - 62·9	26	*57·8*	*39·3 - 76·2*	30	*60·0*	*42·9 - 77·2*
2005-2009	6			22	**68·0**	48·7 - 87·2	6			14	**53·6**	29·8 - 77·5	23	*57·1*	*38·6 - 75·7*	25	*75·8*	*59·2 - 92·4*
Switzerland *																		
1995-1999	5			15	**60·0**	36·3 - 83·8	11	**54·6**	27·0 - 82·2	11	**45·5**	18·1 - 72·9	22	*54·6*	*34·5 - 74·8*	20	*55·1*	*34·0 - 76·1*
2000-2004	4			15	**52·6**	28·1 - 77·0	9			15	**40·0**	16·6 - 63·5	28	**58·4** [R]	44·5 - 72·4	15	*45·8*	*21·5 - 70·0*
2005-2009	2			15	**68·1**	43·1 - 93·1	10	**75·8**	53·0 - 98·5	7			22	*75·1*	*58·1 - 92·1*	12	*74·1*	*50·0 - 98·2*
United Kingdom *																		
1995-1999	57	**61·6**	49·1 - 74·1	126	**64·1**	55·7 - 72·4	93	**62·0**	52·1 - 71·8	95	**50·6**	40·6 - 60·6	204	**60·1**	53·2 - 66·9	167	**56·7**	48·6 - 64·8
2000-2004	56	**57·3**	44·5 - 70·2	119	**72·3**	64·3 - 80·3	91	**66·6**	56·9 - 76·3	124	**62·7**	54·2 - 71·2	213	**66·0**	59·4 - 72·6	177	**65·1**	57·9 - 72·3
2005-2009	50	**56·7**	43·8 - 69·6	123	**70·0**	62·0 - 78·1	85	**73·0**	63·6 - 82·4	110	**65·3**	56·2 - 74·5	196	**67·4**	60·6 - 74·2	172	**69·4**	62·4 - 76·4
**OCEANIA**																		
Australian registries																		
1995-1999	14	**35·8**	13·1 - 58·5	41	**48·8**	33·8 - 63·8	41	**56·1**	41·2 - 71·0	29	**58·7**	41·1 - 76·2	61	**50·9** [R]	41·1 - 60·6	64	**55·4**	43·5 - 67·4
2000-2004	23	**60·4**	40·7 - 80·1	54	**74·0**	62·4 - 85·6	39	**71·8**	57·9 - 85·7	54	**70·4**	58·4 - 82·5	94	**72·5**	62·7 - 82·2	76	**69·4**	59·3 - 79·6
2005-2009	21	**76·1**	58·1 - 94·1	47	**85·5**	75·6 - 95·4	20	**59·3**	41·4 - 77·2	41	**63·2**	49·1 - 77·3	65	**74·2** [R]	65·7 - 82·8	64	**67·4**	56·7 - 78·1
New Zealand *																		
1995-1999	8			14	**78·6**	58·0 - 99·3	11	**54·6**	27·0 - 82·2	9			20	*55·1*	*34·0 - 76·1*	22	*81·9*	*66·2 - 97·6*
2000-2004	4			16	**81·3**	62·8 - 99·8	8			16	**62·6**	39·8 - 85·4	19	*73·7*	*54·5 - 93·0*	25	*64·1*	*45·7 - 82·5*
2005-2009	4			13	**77·6**	56·2 - 99·0	6			11	**75·3**	52·0 - 98·5	15	*64·2*	*42·0 - 86·3*	19	*83·2*	*66·2 - 100·0*
